# Self‐Sustaining Piezozyme Platform for Antifouling via Mechanically Triggered Enzymatic Cascade

**DOI:** 10.1002/advs.202519278

**Published:** 2026-02-20

**Authors:** Jingyu Wang, Yanjun Yu, Rongxin Su, Jiangjiexing Wu

**Affiliations:** ^1^ State Key Laboratory of Chemical Engineering Tianjin Key Laboratory of Membrane Science and Desalination Technology, School of Chemical Engineering and Technology Tianjin University Tianjin China; ^2^ Technical Center for Safety of Industrial Products of Tianjin Customs Tianjin China; ^3^ Key Laboratory of Ocean Observation Technology of Ministry of Natural Resources School of Marine Science and Technology Tianjin University Tianjin China

**Keywords:** marine antifouling, nanozyme, piezocatalytic H_2_O_2_ generation, reactive oxygen species cascade, self‐powered catalysis

## Abstract

Harnessing ubiquitous mechanical energy for catalytic activation offers an emerging pathway toward self‐sustaining environmental protection systems. Herein, we report a universal self‐powered piezozyme platform that converts low‐frequency mechanical stimuli into an enzymatic cascade, enabling chemical‐free marine antifouling. The system is constructed from a CeO_2_/g‐C_3_N_4_ heterostructure, where piezoelectric polarization in g‐C_3_N_4_ drives electron‐hole separation and oxygen reduction to generate H_2_O_2_ in situ, while CeO_2_ nanophases initiate dual haloperoxidase‐ and peroxidase‐like enzymatic reactions, yielding potent reactive oxygen species (•OH and HOBr). Unlike conventional photocatalytic or sacrificial approaches, this mechanically triggered cascade continuously operates under wave motion without external light or chemical input. Beyond antibacterial testing (>99.99% inactivation), it represents the first piezocatalytic nanozyme coating validated in a 180‐day marine field test, maintaining <5% biofouling coverage. This proposed self‐sustaining catalytic strategy not only offers an environmentally benign antifouling solution but also opens new opportunities for the design of wave‐driven catalytic systems in marine protection and sustainable energy–environmental applications.

## Graphical abstract

Wave‐powered CeO_2_/g‐C_3_N_4_ piezozyme coating converts ocean motion into an enzymatic H_2_O_2_‐to‐ROS cascade, realizing 180‐day eco‐friendly marine antifouling.

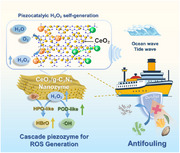

## 1 Introduction

Marine biofouling, the undesired accumulation of microorganisms and macroorganisms on submerged surfaces, imposes significant operational and environmental burdens in marine industries [1, 2, 3, 4]. Early antifouling strategies relied on copper‐containing or organic biocides, which are effective short‐term but pose ecological risks and lose efficacy over time [5, 6]. Physical coatings, such as low‐surface‐energy or fouling‐release surfaces, reduce chemical toxicity but are sensitive to environmental conditions [7, 8]. More recently, green alternatives including natural products, antifouling enzymes, and small‐molecule antibacterial agents have been explored. These methods improve biocompatibility but often have limited stability and short lifetimes, restricting their effectiveness in dynamic marine environments [9, 10]. In recent years, nanozymes, nanomaterials with enzyme‐like catalytic activities [11, 12, 13, 14], have emerged as environmentally benign alternatives due to their high stability, facile synthesis, and catalytic versatility [15, 16, 17]. However, their catalytic efficiency in marine environments is fundamentally constrained by the extremely low natural concentration of hydrogen peroxide (H_2_O_2_) in seawater, typically in the range of 100–250 nm [18, 19]. Since H_2_O_2_ is a crucial substrate for reactive oxygen species (ROS)‐mediated antimicrobial pathways, its scarcity limits ROS generation and thus hampers the practical utility of nanozymes for antifouling [20, 21, 22].

Several strategies have been explored to address this limitation, including photocatalytic [23, 24], electrocatalytic [25, 26], and sacrificial chemical approaches [27, 28, 29] for in situ H_2_O_2_ generation. However, these methods face intrinsic challenges such as light dependence, external energy input, or long‐term material instability. Piezoelectric catalysis, which enables the conversion of mechanical energy into chemical reactivity via mechanically induced polarization [30, 31], offers a compelling alternative for light‐independent, self‐powered catalysis in dynamic marine environments [32]. Piezoelectric materials can generate internal electric fields upon deformation, facilitating charge separation and promoting oxygen reduction reactions to yield H_2_O_2_ and other ROS [33, 34, 35]. This mechanism is particularly attractive for autonomous antifouling systems, as mechanical stimuli such as wave motion and current turbulence are ubiquitous and persistent in the ocean [36, 37]. Although the discovery of piezocatalysis‐enabled H_2_O_2_ generation is a breakthrough, the development of high‐performance self‐powered piezozymes and their practical application in marine antifouling remains largely unexplored.

Graphitic carbon nitride (g‐C_3_N_4_) exhibits notable piezoelectric performance due to its non‐centrosymmetric layered structure, enabling efficient conversion of mechanical energy into chemical energy under mechanical stimulation and catalyzing the oxygen reduction reaction to generate H_2_O_2_ [38, 39]. In addition, g‐C_3_N_4_ possesses excellent chemical stability and environmental tolerance, making it an ideal piezoelectric component for self‐powered marine antifouling systems. Nevertheless, its weak enzyme‐like catalytic activity makes it inefficient at transforming H_2_O_2_ into highly biocidal ROS, limiting its standalone antifouling potential [40]. CeO_2_, a widely studied nanozyme, has been demonstrated to exhibit multiple enzyme‐like activities, showing great potential for catalyzing ROS generation and achieving antibacterial and antifouling effects [41, 42]. In particular, its haloperoxidase (HPO)‐like activity can utilize halide ions in seawater (e.g., Br^−^) to catalyze the formation of strongly oxidative hypohalous species (e.g., HOBr), a feature especially suitable for controlling biofouling in marine environments [43, 44]. Nevertheless, existing CeO_2_‐based antifouling systems heavily rely on the external addition of H_2_O_2_, limiting their sustained application in real seawater [45].

**FIGURE 1:**
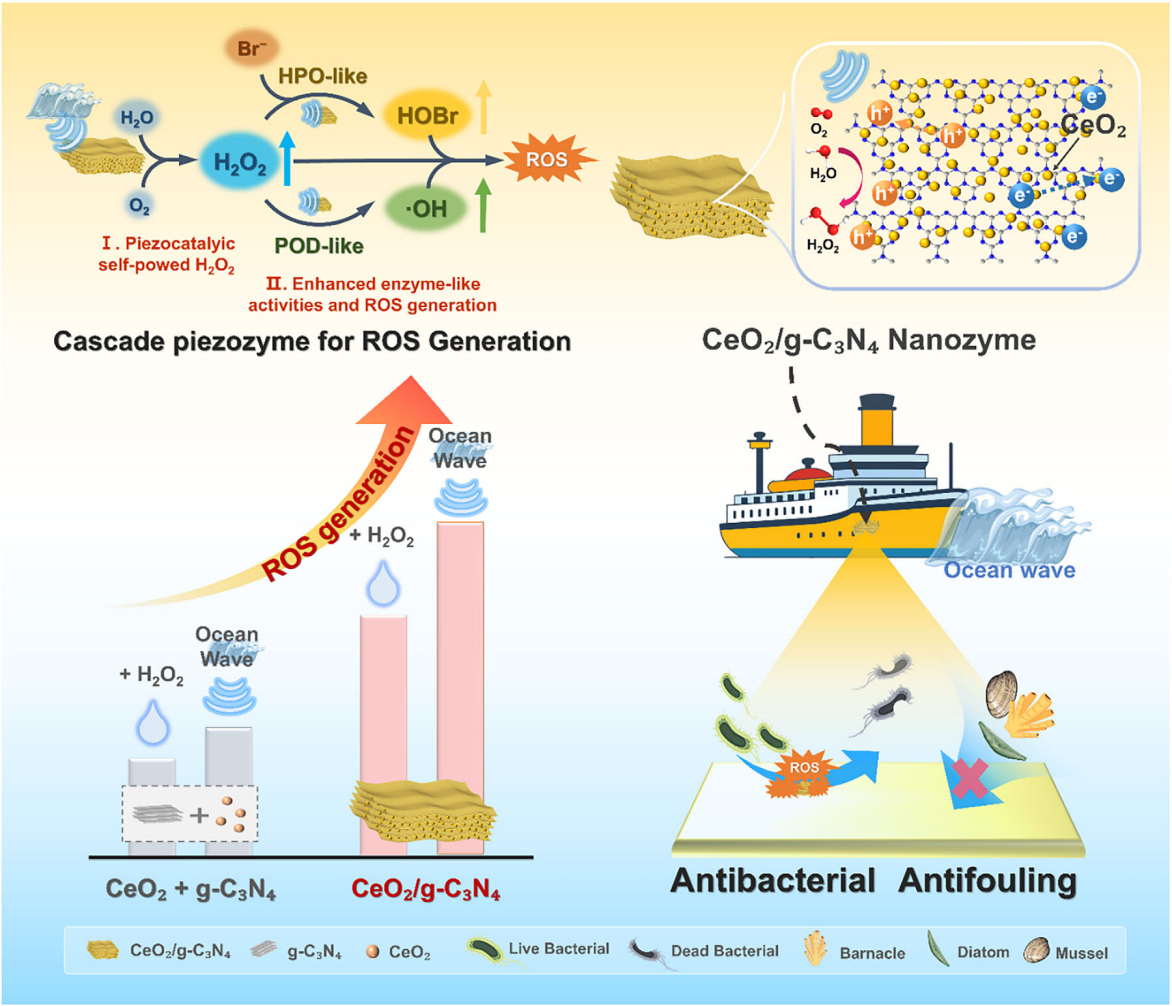
Schematic diagram of the piezoelectric self‐powered CeO_2_/g‐C_3_N_4_ cascade nanozyme for antibacterial and antifouling applications.

Herein, inspired by the cascade architecture of natural enzymatic systems, where upstream oxidases generate H_2_O_2_ for downstream peroxidase reactions, we constructed an artificial enzyme cascade capable of self‐supplying H_2_O_2_ under mechanical stimulation. In this design, redox‐active CeO_2_, a prototypical nanozyme with excellent HPO‐ and peroxidase (POD)‐like activities, was integrated with g‐C_3_N_4_ to form a mechanically activated piezozyme (Figure 1). Upon low‐frequency mechanical disturbance (e.g., ocean waves), the piezoelectric response of g‐C_3_N_4_ triggers in situ H_2_O_2_ formation, which is subsequently utilized by CeO_2_ to generate potent oxidative species such as •OH and HOBr via enzyme‐like pathways. Beyond the cascade mechanism, the built‐in interfacial electric field promotes charge transfer, Ce^3+^/Ce^4+^ redox cycling, and defect activation, further enhancing catalytic efficiency. This bioinspired, self‐powered piezozyme system not only achieves outstanding antibacterial activity but also demonstrates long‐term antifouling efficacy in marine field tests, providing an innovative and feasible solution for the development of scalable and environmentally benign next‐generation antifouling coatings.

## 2 Results and Discussion

### 2.1 Preparation and Characterization of CeO_2_/g‐C_3_N_4_ Nanozyme

**FIGURE 2:**
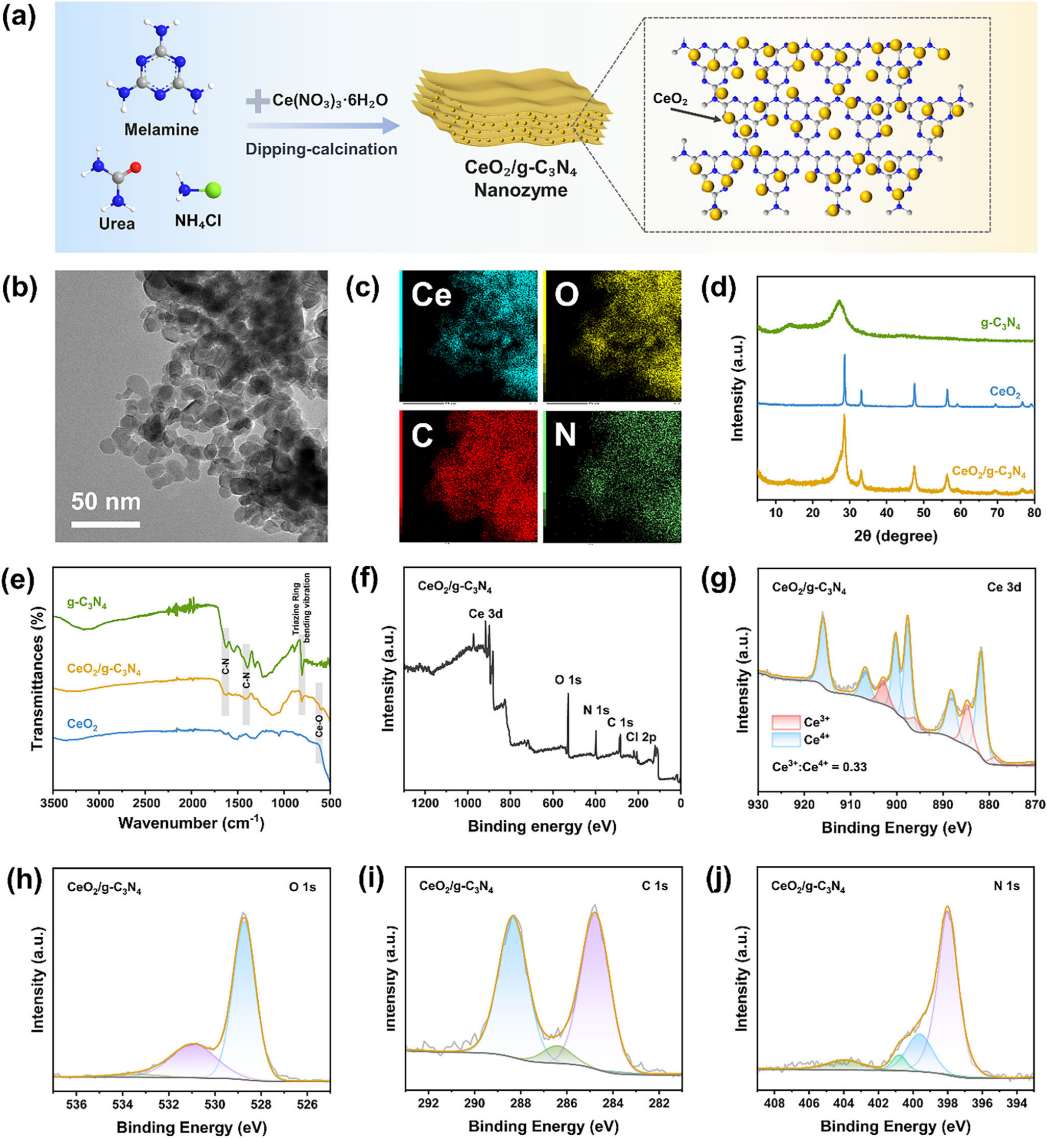
Preparation and characterization of CeO_2_/g‐C_3_N_4_ nanozyme. a) Schematic representation for the synthesis of CeO_2_/g‐C_3_N_4_ nanozyme. b) TEM image and c) EDS mapping of CeO_2_/g‐C_3_N_4_. d) XRD patterns and e) FT‐IR spectra of g‐C_3_N_4_, CeO_2_, and CeO_2_/g‐C_3_N_4_. f) The XPS survey spectra, g) the Ce 3d, h) the O 1s, i) the C 1s, j) the N 1s.

As shown in Figure 2a, the CeO_2_/g‐C_3_N_4_ nanozyme was synthesized via an improved dipping‐calcination strategy. A precursor solution containing Ce(NO_3_)_3_·6H_2_O, urea, melamine, and NH_4_Cl was ultrasonically mixed, followed by impregnation and evaporation. Subsequent calcination yielded the final light‐yellow composite nanozyme (Figure S1). Transmission electron microscopy (TEM) was employed to investigate the morphology and microstructure of the CeO_2_/g‐C_3_N_4_ composite (Figure 2b). The results revealed a typical layered structure derived from g‐C_3_N_4_, with CeO_2_ nanoparticles uniformly anchored on the nanosheets, indicating successful composite formation. The corresponding Energy‐dispersive X‐ray spectroscopy (EDS) mapping (Figure 2c) showed that Ce and O elements were homogeneously distributed and co‐localized with the C and N elements of the g‐C_3_N_4_ matrix, confirming the intimate interfacial contact between the two components. Additionally, TEM and high‐resolution TEM images presented in Figure S2 reveal the nanoscale morphology of the composite and the intimate contact between CeO_2_ and g‐C_3_N_4_. The observed lattice fringes with interplanar spacings corresponding to the (110), (100), and (111) planes of CeO_2_ further confirm its well‐defined crystalline structure. X‐ray diffraction (XRD) patterns of g‐C_3_N_4_, CeO_2_, and CeO_2_/g‐C_3_N_4_ are shown in Figure 2d. Pure g‐C_3_N_4_ exhibits characteristic peaks at 13.4° and 27.2°, corresponding to the (100) lattice plane of triazine units and the (002) plane of interlayer aromatic stacking, respectively. CeO_2_ displays peaks at 28.5°, 33.1°, 47.5°, and 56.3°, indexed to the (111), (200), (220), and (311) planes of cubic fluorite‐phase CeO_2_ (JCPDS 34–0394). The XRD pattern of the CeO_2_/g‐C_3_N_4_ nanozyme shows a weak peak at 13.4°, corresponding to the (100) plane of g‐C_3_N_4_, and a broadened peak around 27.8°, which is slightly shifted to lower angles compared with pure CeO_2_. This broadening and shift likely result from the overlap of reflections from both g‐C_3_N_4_ and CeO_2_. Additional peaks corresponding to the (111), (200), (220), and (311) planes of CeO_2_ are also observed. Collectively, these results indicate that both CeO_2_ and g‐C_3_N_4_ are present in the composite, with the layered structure of g‐C_3_N_4_ preserved. Complementing this, the ^13^C CP/MAS NMR spectrum of CeO_2_/g‐C_3_N_4_ nanozyme (Figure S3) shows peaks at ∼156 and 164 ppm, corresponding to the triazine rings and amino linkages of g‐C_3_N_4_, confirming that its chemical structure remains intact. Fourier transform infrared (FT‐IR) spectroscopy (Figure 2e) revealed structural details of the materials. Pure g‐C_3_N_4_ displayed a strong peak at 807 cm^−1^ (out‐of‐plane bending of heptazine rings) and multiple peaks between 1230–1640 cm^−1^ (C‐N heterocyclic skeletal vibrations). Broad peaks at 3300–3500 cm^−1^ were attributed to uncondensed amino groups (–NH/–NH_2_) and adsorbed water. CeO_2_ exhibited characteristic metal‐oxygen vibrations at 400–750 cm^−1^, with a sharp peak at 624 cm^−1^ (O─Ce─O symmetric stretching). Consistent with the FT‐IR analysis, the Raman spectra (Figure S4) confirmed the coexistence of CeO_2_ and g‐C_3_N_4_ in the composite and the preservation of their respective structural features. In addition, thermogravimetric analysis (TGA) was performed to quantitatively determine the g‐C_3_N_4_ content in the CeO_2_/g‐C_3_N_4_ nanozyme (Figure S5). A slight weight loss below 100°C was observed, which is attributed to the desorption of physically adsorbed moisture and surface hydroxyl species. A pronounced mass loss between ∼500°C and 650°C corresponds to the thermal decomposition of g‐C_3_N_4_, while the residual mass above 650°C (12.54 wt.%) is assigned to thermally stable CeO_2_. Based on this analysis, the CeO_2_ content in the composite is estimated to be approximately 15 wt.%, with g‐C_3_N_4_ constituting the remaining fraction of the composite.

X‐ray photoelectron spectroscopy (XPS) analysis confirmed the elemental composition of the CeO_2_/g‐C_3_N_4_ composite, detecting cerium, oxygen, carbon, nitrogen, and trace chlorine likely originating from residual NH_4_Cl (Figure 2f). As shown in Figure 2g, due to the non‐stoichiometric nature of cerium and the multiple d‐orbital splitting, cerium exists in a mixed oxidation state of Ce^3+^/Ce^4+^. The peaks located at 881.79, 888.18, 897.64, 900.23, 906.80, and 915.93 eV are attributed to Ce^4+^, while those at 878.95, 884.76, 896.23, and 902.93 eV correspond to Ce^3+^, indicating the coexistence of Ce^3+^ and Ce^4+^ on the sample surface with an approximate ratio of Ce^3+^: Ce^4+^ = 0.33:1. In this scenario, certain lattice oxygen atoms must escape from the fluorite structure to maintain the charge neutrality of CeO_2_, leading to the formation of intrinsic oxygen vacancies. This finding is further corroborated by the high‐resolution O 1s spectrum (Figure 2h). The O 1s spectrum is deconvoluted into three distinct components at 528.72 eV (lattice oxygen), 530.92 eV (defective or adsorbed oxygen species), and 533.37 eV (hydroxyl groups and/or carbonates). As shown in the carbon spectrum (Figure 2i), two distinct chemical states are observed. The peak at 288.28 eV corresponds to hybridized carbon atoms within the sp^2^‐triazine structural units (N─C═N), while the characteristic peak at 284.80 eV is attributed to graphitic carbon (C─C/C═C). The core species of N 1s, as depicted in Figure 2j, exhibit four distinct peaks, including a *π*–π* electronic transition at approximately 404.08 eV present in all samples, along with graphitic nitrogen, pyrrolic nitrogen, and pyridinic nitrogen. Collectively, the above structural and compositional analyses confirm the successful synthesis of the CeO_2_/g‐C_3_N_4_ composite nanozyme.

### 2.2 Piezocatalytic H_2_O_2_ Generation

**FIGURE 3:**
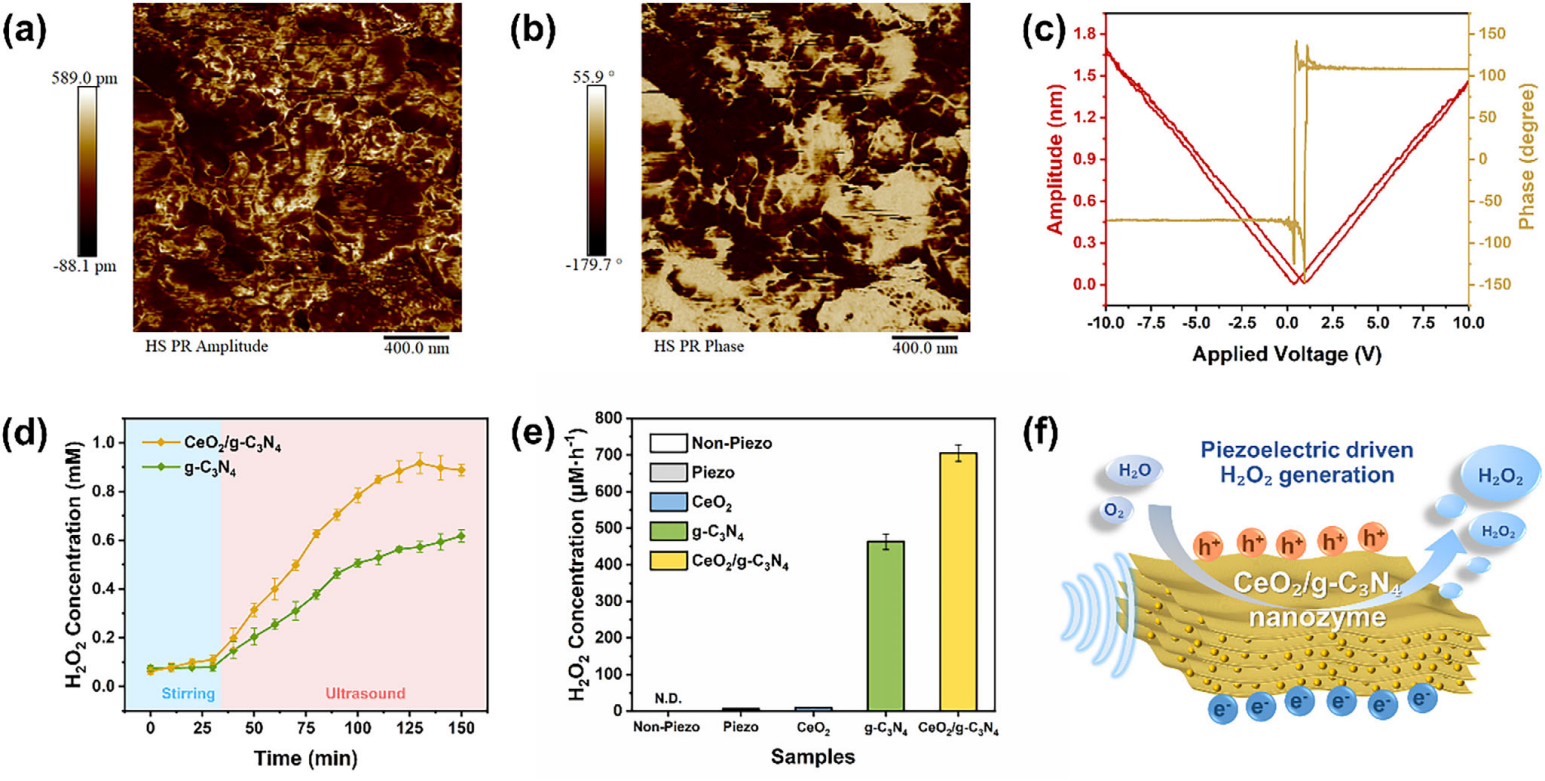
Piezoelectric properties and piezocatalytic H_2_O_2_ generation of CeO_2_/g‐C_3_N_4_ nanozyme. a) Amplitude map, b) phase diagram, and c) piezoresponsive amplitude and phase curves of CeO_2_/g‐C_3_N_4_. d) Time‐accumulated H_2_O_2_ amount curve by piezocatalysis of g‐C_3_N_4_ and CeO_2_/g‐C_3_N_4_ under piezo‐activation. e) Comparison of piezocatalytic H_2_O_2_ production efficiency in different samples (None detected was denoted as N.D.). Error bars indicate standard deviations of three independent measurements. f) Schematic diagram of piezoelectric‐driven H_2_O_2_ generation over CeO_2_/g‐C_3_N_4_.

Following the successful synthesis and structural characterization of the CeO_2_/g‐C_3_N_4_ nanozyme, we first investigated its piezoelectric properties using piezoresponse force microscopy (PFM). In this experiment, an alternating voltage was applied through a conductive cantilever tip in contact mode to induce piezoelectric surface oscillations. Figure S6 presents representative PFM morphology images. Within a randomly selected 2 × 2 µm^2^ scanning area, the sample height varied between −129.5 and 113.9 nm. The relative amplitude and phase switching signals observed in Figure 3a,b exhibit distinct color contrasts, clearly confirming the presence of a piezoelectric response in CeO_2_/g‐C_3_N_4_. The pronounced phase signal variations reflect localized polarization directions across multiple domains (Figure 3c). Upon applying a 10 V AC bias between the tip and sample, the local polarization‐electric field hysteresis loop exhibited significant hysteresis and 180° phase reversal, indicative of reversible piezoelectric domain switching under an external electric field. A well‐defined butterfly‐shaped amplitude‐voltage curve further corroborated the piezoelectric behavior of CeO_2_/g‐C_3_N_4_. Compared to the piezoelectric coefficient (*d_33_*) of pure g‐C_3_N_4_ (14.89 pm V^−1^, Figure S7), the CeO_2_/g‐C_3_N_4_ nanozyme exhibited an enhanced *d_33_* value of 19.64 pm V^−1^. These results confirm the enhanced piezoelectric performance of the CeO_2_/g‐C_3_N_4_, which is attributed to the intensified interfacial polarization within the heterojunction, leading to a greater piezoelectric potential response.

Building on this confirmation of piezoelectricity, we systematically evaluated the piezocatalytic performance of the CeO_2_/g‐C_3_N_4_ nanozyme. The H_2_O_2_ concentration was quantified using a standard calibration curve established in Figure S8. In this study, ultrasonic stimulation was employed to mimic marine hydrodynamic forces such as waves and tides, serving as an energy input to trigger piezo‐activation. As shown in Figure 3d, the initial 30 min of dark stirring allowed the catalyst to reach adsorption‐desorption equilibrium, indicating that H_2_O_2_ production was negligible in the absence of piezoelectric stimulation. However, upon piezo‐activation, the H_2_O_2_ concentration significantly increased, demonstrating that the piezoelectric effect serves as the primary driving force for catalytic enhancement. Compared to pristine g‐C_3_N_4_, the CeO_2_/g‐C_3_N_4_ nanozyme exhibited a markedly higher H_2_O_2_ yield, underscoring the synergistic effect between CeO_2_ and g‐C_3_N_4_ in boosting catalytic performance. In the control experiments (Figure 3e), the sonochemical event without the introduction of catalysts led to an H_2_O_2_ production rate of 6.7 µmol·h^−1^, which was attributed to the combination of •OH radicals generated from H–O bond cleavage of H_2_O molecules through cavitation effects. Without piezo‐activation, no detectable H_2_O_2_ was produced. Additionally, CeO_2_ alone exhibited negligible catalytic activity. Notably, the CeO_2_/g‐C_3_N_4_ hybrid demonstrated exceptional H_2_O_2_ generation efficiency with a production rate of 705 µm h^−1^, achieving a 1.52‐fold improvement compared to the 463 µm h^−1^ rate observed for pure g‐C_3_N_4_. The underlying mechanism can be attributed to localized electric fields generated within the g‐C_3_N_4_ matrix under mechanical stimulation, which facilitate charge separation and accelerate electron transfer to surface‐adsorbed O_2_. CeO_2_ further promotes oxygen activation and interfacial charge transfer, leading to superior piezocatalytic efficiency (Figure 3f).

### 2.3 Piezoelectric Enhanced Enzyme‐Like Activities

**FIGURE 4:**
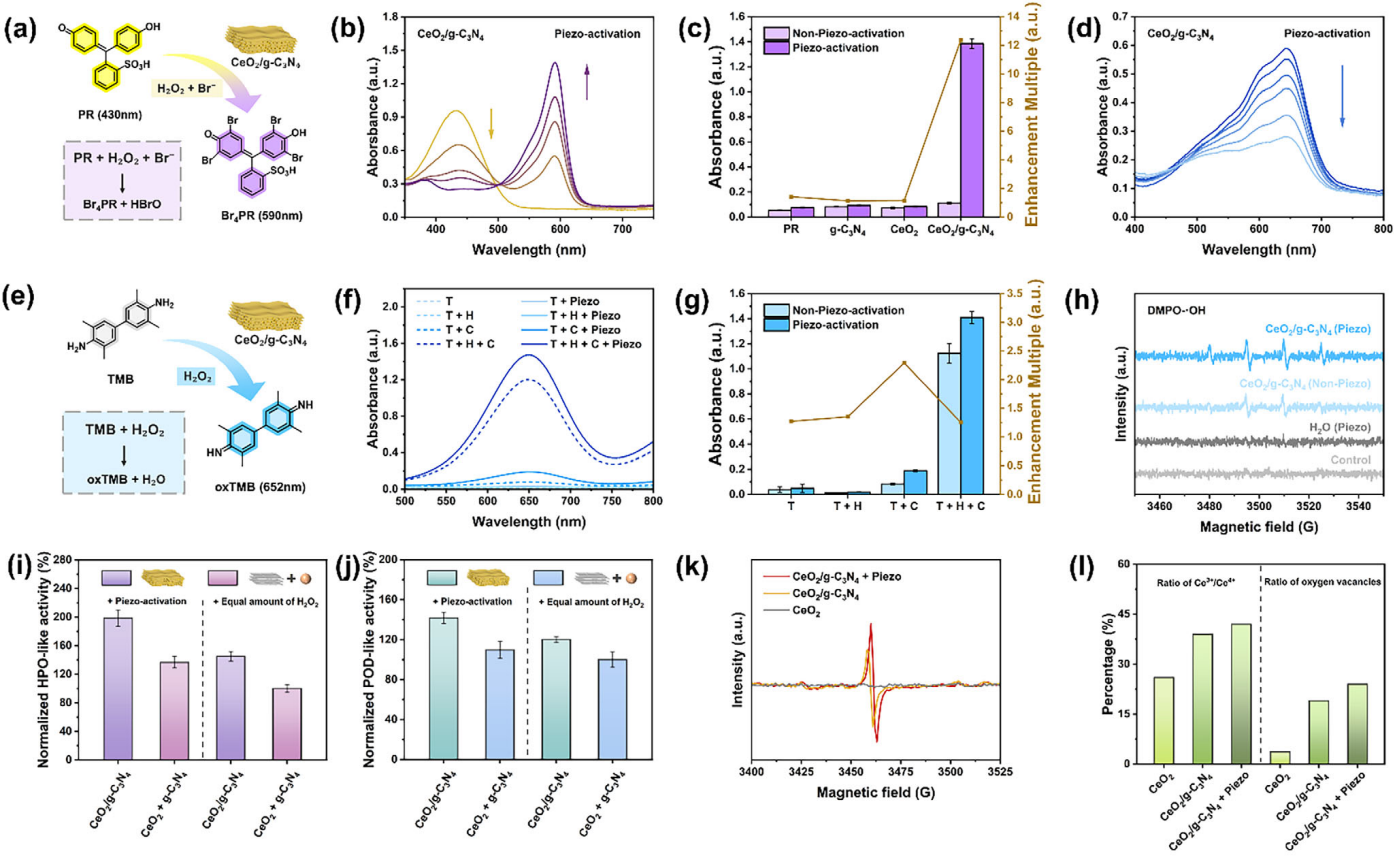
Piezoelectric enhanced HPO‐ and POD‐like activities of CeO_2_/g‐C_3_N_4_ nanozyme and mechanistic insights. a) Schematic mechanism of PR assay. b) Time‐dependent UV–vis spectra illustrating the piezoelectric‐enhanced HPO‐like activities of CeO_2_/g‐C_3_N_4_ without H_2_O_2_. c) Comparative analysis of HPO‐like activities across experimental groups under piezoelectric activation and non‐piezoelectric conditions, without H_2_O_2_ supplementation. d) The time‐dependent degradation curve of celestine blue was catalyzed by CeO_2_/g‐C_3_N_4_ under piezoelectric activation. e) Schematic mechanism of the TMB oxidation reaction. f) Piezoelectric enhanced POD‐like activities of different groups in the absence and presence of piezoelectric activation. g) Multiple piezoelectric enhancements under different conditions. h) EPR spectra of •OH trapped by DMPO. i) HPO‐ and j) POD‐like activities of CeO_2_/g‐C_3_N_4_ and the physical mixture of CeO_2_ + g‐C_3_N_4_ under piezoelectric activation (left) and under externally added H_2_O_2_ without piezo‐activation (right). All activities were normalized to that of the physical mixture under H_2_O_2_ supplementation. k) EPR spectra of oxygen vacancies in CeO_2_, CeO_2_/g‐C_3_N_4_, and CeO_2_/g‐C_3_N_4_ under piezoelectric activation. l) Comparison of Ce valence states and oxygen vacancy contents in CeO_2_ and CeO_2_/g‐C_3_N_4_ before and after piezoelectric activation.

Inspired by the piezoelectric H_2_O_2_ generation capability of the CeO_2_/g‐C_3_N_4_ nanozyme, we further evaluated its HPO‐like activity using phenol red (PR) as the substrate (Figure 4a). Upon halogenation by HOBr, PR undergoes a color change from yellow to blue‐violet, corresponding to a decrease in absorbance at 434 nm and an increase at 590 nm due to the formation of brominated PR (Br_4_PR). As shown in Figure 4b, in the absence of externally supplied H_2_O_2_, the characteristic absorption peak of PR at λ ≈ 590 nm was significantly enhanced under piezo‐activation, indicating that the nanozyme facilitated in situ H_2_O_2_ generation, which subsequently triggered oxidative halogenation reactions, demonstrating HPO‐like activity. To further quantify the catalytic activity under different conditions, we analyzed the absorbance changes at 590 nm. When CeO_2_/g‐C_3_N_4_ was introduced, a pronounced increase in absorbance at 590 nm was observed, highlighting its superior enzyme‐mimicking performance. Notably, under piezo‐activation and without external H_2_O_2_ addition, the CeO_2_/g‐C_3_N_4_ nanozyme exhibited a 12‐fold enhancement in catalytic activity compared to the non‐piezo‐activated condition (Figure 4c). This enhancement shows that piezoelectrically generated H_2_O_2_ drives efficient HPO‐like activity, eliminating the need for an external H_2_O_2_ supply. Even in the presence of externally added H_2_O_2_ (Figure S9), mechanical stimulation still significantly enhanced the HPO‐like activity of the CeO_2_/g‐C_3_N_4_ nanozyme. However, when g‐C_3_N_4_ or CeO_2_ was used individually as the catalyst, no significant piezoelectric‐driven enhancement in absorbance was observed. Specifically, g‐C_3_N_4_ showed no catalytic activity either with or without ultrasonic irradiation. Although CeO_2_ exhibited certain intrinsic catalytic activity, no further increase in absorbance occurred upon piezoelectric activation, indicating the absence of piezocatalytic behavior in CeO_2_ without externally added H_2_O_2_ (Figure S10). Additionally, CB selectively reacts with the HOBr intermediate in HPO‐like reactions, leading to a decrease in absorbance at 640 nm. In the absence of added H_2_O_2_, CeO_2_/g‐C_3_N_4_ nanozymes under piezo‐activation induced a 15‐fold reduction in absorbance at 640 nm, further confirming the efficient formation of HOBr (Figure 4d).

Moreover, the POD‐like activity of CeO_2_/g‐C_3_N_4_ was evaluated using TMB as the substrate. The catalytic decomposition of H_2_O_2_ generates •OH radicals, which oxidize TMB, resulting in a characteristic absorption peak at 652 nm (Figure 4e). As shown in Figure 4f, the UV–vis absorption spectra of CeO_2_/g‐C_3_N_4_ + H_2_O_2_, CeO_2_/g‐C_3_N_4_ alone, H_2_O_2_ alone, and TMB alone (under both piezo‐activated and non‐piezo‐activated conditions) were recorded. Notably, CeO_2_/g‐C_3_N_4_ exhibited significantly enhanced POD‐mimicking activity under piezo‐activation. Figure 4g quantitatively illustrates the piezoelectric enhancement effect on the POD‐like activity of CeO_2_/g‐C_3_N_4_ compared to other groups. In the absence of H_2_O_2_, mechanical stimulation had no impact on TMB oxidation, confirming the stability of TMB under ultrasonic conditions. When both H_2_O_2_ and TMB were present, the absorbance change remained minimal, indicating a limited enhancement effect of mechanical energy on H_2_O_2_ alone. The addition of CeO_2_/g‐C_3_N_4_ led to an increase in absorbance, which further rose by over 25% under piezoelectric activation, indicating enhanced POD‐like activity. Electron paramagnetic resonance (EPR) analysis was conducted to investigate the generation of •OH during the POD‐like catalytic process of CeO_2_/g‐C_3_N_4_. Figure 4h shows the EPR spectra obtained in the presence of the spin‐trapping agent 5,5‐dimethyl‐1‐pyrroline N‐oxide (DMPO), with and without piezo‐activation. The characteristic DMPO–•OH (1:2:2:1) signal was clearly detected, confirming the production of hydroxyl radicals as a key intermediate in the catalytic reaction. Under piezo‐activation, the intensity of the DMPO–•OH signal at the peak located at ∼3510 G exhibited a 1.3‐fold increase compared to the non‐piezo‐activated condition, providing direct evidence that mechanical stimulation enhances the POD‐like activity of CeO_2_/g‐C_3_N_4_ by promoting in situ •OH generation. Taken together, these results demonstrate that the CeO_2_/g‐C_3_N_4_ nanozyme possesses dual HPO‐ and POD‐like activities, both of which are significantly boosted by piezoelectric activation through in situ ROS generation.

To elucidate the origin of the enhanced catalytic activity observed in the CeO_2_/g‐C_3_N_4_ nanozyme, two sets of control experiments were conducted. As shown in Figure 4i,j, under identical piezoelectric activation conditions, the CeO_2_/g‐C_3_N_4_ composite nanozyme exhibited significantly higher HPO‐ and POD‐like activities compared to the physical mixture of CeO_2_ and g‐C_3_N_4_. This result indicates that the integrated nanozyme facilitates the efficient transfer of reactive intermediates such as H_2_O_2_ and bromide ions, thereby enabling a cascade catalytic process and minimizing intermediate consumption and degradation. This synergistic effect was not observed in the physically mixed system. To further clarify whether the catalytic enhancement is solely attributable to the in situ generation of H_2_O_2_, an additional control experiment was designed by externally supplying the same amount of H_2_O_2_ produced under ultrasonic conditions but without applying piezoelectric stimulation. Even at the same H_2_O_2_ concentration, the piezoelectrically activated CeO_2_/g‐C_3_N_4_ nanozyme exhibited markedly higher catalytic activity. This suggests that the piezoelectric field itself contributes positively to the catalytic process, possibly by promoting charge separation, activating substrate molecules, or stabilizing reactive species at the solid–liquid interface. These findings collectively demonstrate that the superior enzyme‐mimetic activity of CeO_2_/g‐C_3_N_4_ arises from a synergistic mechanism involving intimate interfacial contact that enables a cascade effect and piezoelectric activation that enhances both ROS generation.

To further elucidate the intrinsic mechanism behind the enhanced catalytic activity of the CeO_2_/g‐C_3_N_4_ nanozyme under piezoelectric stimulation, we systematically conducted XPS and EPR analyses to investigate its valence state distribution and defect structure. As shown in Figure S11, compared with pristine CeO_2_, the CeO_2_/g‐C_3_N_4_ composite exhibits a significantly higher proportion of Ce^3+^ and a stronger defect‐related signal in the O 1s spectrum, indicating that the interfacial interaction within the composite promotes the partial reduction of Ce^4+^ and the formation of oxygen vacancies. On this basis, we further examined the electronic structure changes before and after piezoelectric activation (Figure S12). The results show that ultrasonic excitation slightly increased the Ce^3+^ content and the defect oxygen signal, suggesting that mechanical polarization may further induce valence modulation and defect formation. This trend is also supported by the EPR analysis (Figure 4k), where a more intense signal corresponding to oxygen vacancies was observed after piezoelectric activation. To quantitatively compare the Ce^3+^ content and oxygen vacancy concentration among the three samples, we performed statistical analysis of the XPS data, as summarized in Figure 4l. Compared to CeO_2_, CeO_2_/g‐C_3_N_4_ possesses a higher density of active sites even under static conditions, and piezoelectric stimulation further enhances its catalytic potential. In summary, the superior catalytic performance of CeO_2_/g‐C_3_N_4_ is not only attributed to its intrinsic defect structure but also benefits from the electronic modulation and defect activation induced by the piezoelectric field. These two effects work synergistically to endow the system with excellent ROS generation capability, thereby laying a solid foundation for its antibacterial and antifouling performance.

### 2.4 Antibacterial Performance

**FIGURE 5:**
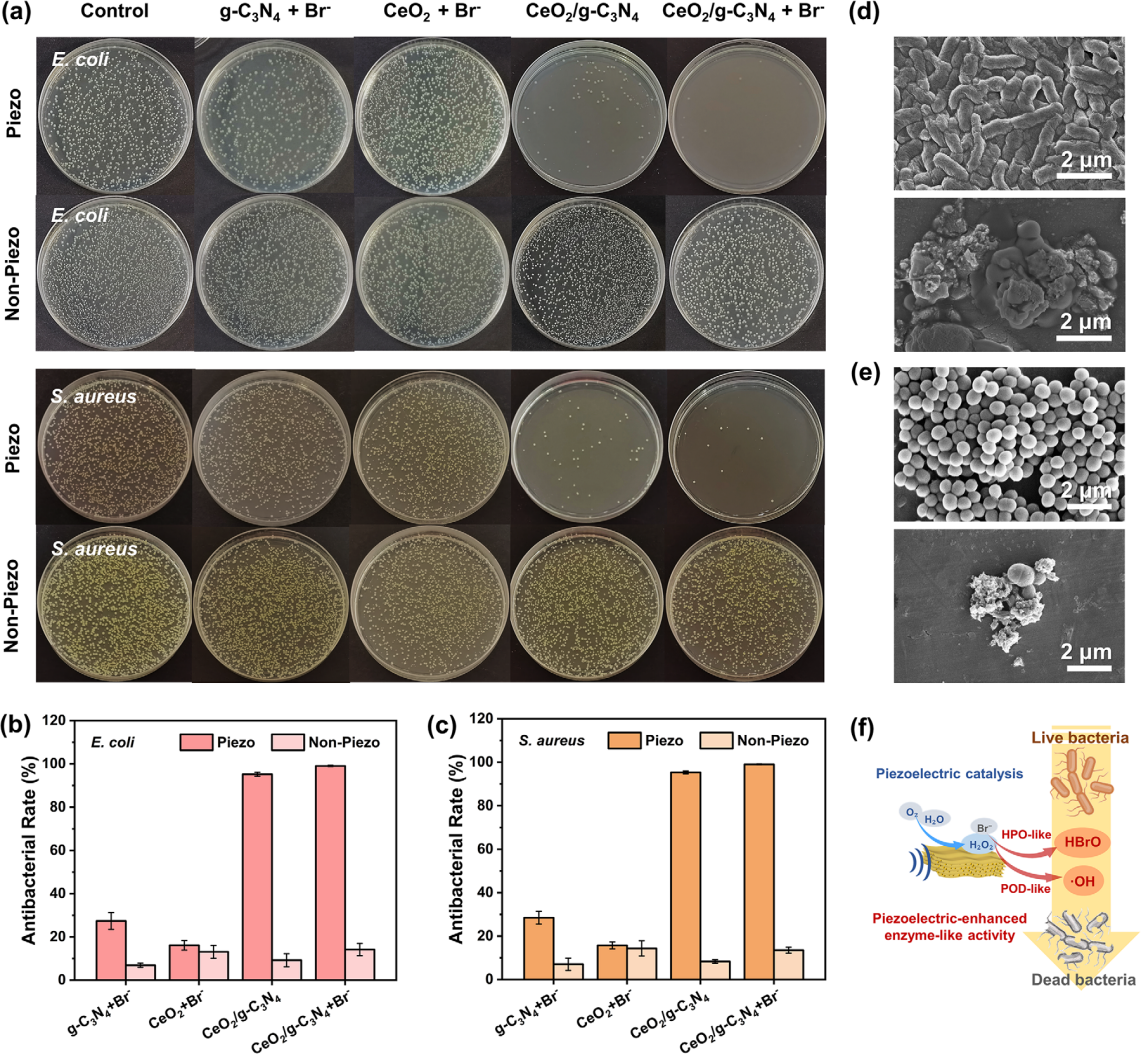
Antibacterial performance of CeO_2_/g‐C_3_N_4_ nanozyme. a) Digital photographs of colonies of *E. coli* and *S. aureus* treated with CeO_2_/g‐C_3_N_4_ + Br^−^ under piezo‐activation, the treatments of blank control, g‐C_3_N_4_ + Br^−^, CeO_2_ + Br^−^, and CeO_2_/g‐C_3_N_4_ were used as controls. Antibacterial rate of b) *E. coli* and c) *S. aureus* treated with piezo‐activation (Piezo) and non‐piezo conditions (Non‐Piezo). SEM images of d) *E. coli* and e) *S. aureus* under piezoelectric conditions comparing control and CeO_2_/g‐C_3_N_4_ + Br^−^ treatment groups. f) Schematic illustration of the possible antibacterial mechanism of CeO_2_/g‐C_3_N_4_. Error bars indicate standard deviations of three independent measurements.

The core challenge in marine antifouling lies in inhibiting the initial colonization of bacteria on ship hulls or equipment surfaces. To assess the antibacterial activity of CeO_2_/g‐C_3_N_4_ nanozymes, we selected both Gram‐negative *Escherichia coli* (*E. coli*) and Gram‐positive *Staphylococcus aureus* (*S. aureus*) as model organisms. Comparative experiments were conducted between the experimental group (CeO_2_/g‐C_3_N_4_ + Br^−^) and control groups (blank bacterial suspension, g‐C_3_N_4_ + Br^−^, CeO_2_ + Br^−^, and CeO_2_/g‐C_3_N_4_ alone) under both piezo‐activated and non‐piezo conditions. As illustrated in Figure 5a, under static conditions, all groups exhibited limited antibacterial effects, indicating that in the absence of piezoelectric activation or exogenous H_2_O_2_, the materials have negligible bactericidal capacity. Under piezoelectric activation, both g‐C_3_N_4_ + Br^−^ and CeO_2_ + Br^−^ showed only minor reductions in bacterial colonies (Figure S13), whereas CeO_2_/g‐C_3_N_4_ alone achieved over 95% inactivation, attributable to its POD‐like activity. The addition of Br^−^ to the composite further enhanced the antibacterial effect via HPO‐like activity, yielding a kill rate exceeding 99% (Figure 5b,c). Further confirmation was provided by SEM images, which revealed significant damage to bacterial membranes in the CeO_2_/g‐C_3_N_4_ + Br^−^ group after piezoelectric treatment (Figure 5d,e). Compared to the control group, both *E. coli* and *S. aureus* exhibited membrane rupture, deformation, and structural collapse, indicating compromised cell integrity. To further mimic the trace H_2_O_2_ naturally present in seawater, we introduced 250 nm H_2_O_2_ into the reaction system (Figure S14). Under piezoelectric activation, the H_2_O_2_ + Br^−^ control group exhibited only a slight decrease in bacterial colonies, likely due to ultrasonic cavitation, indicating that the natural concentrations of these species in seawater are insufficient for significant bacterial inactivation. In this case, CeO_2_/g‐C_3_N_4_ + Br^−^ + H_2_O_2_ under piezoelectric activation achieved >99.99% inactivation of both bacterial strains, corresponding to a 10 000‐fold reduction in viable colonies. These findings confirm the synergistic effect of POD‐ and HPO‐like activities activated by piezoelectric input, highlighting the nanozyme's strong potential for preventing early‐stage bacterial colonization in marine environments. To gain deeper insight into the dynamic cascade underlying the high antibacterial efficiency, we performed time‐resolved sampling within the antibacterial system. At 5, 15, 30, 60, 90, and 120 min, key intermediates (H_2_O_2_, •OH, and HOBr) and antibacterial rate were simultaneously monitored (Figure S15). H_2_O_2_ initially accumulates and then maintains a relatively low steady‐state level, indicating its role as a transient intermediate that is continuously generated and consumed. At the early stage, limited H_2_O_2_ availability and high bacterial density restrict ROS accumulation, resulting in weak ROS signals and a slow increase in antibacterial efficiency. As the reaction proceeds, sustained H_2_O_2_ supply together with decreased bacterial density reduces ROS consumption, enabling the gradual accumulation of •OH and HOBr and leading to a markedly accelerated bacterial inactivation. Overall, these results clearly demonstrate a continuous, self‐sustained cascade from H_2_O_2_ generation to ROS production and effective antibacterial action under mechanical stimulation. To further identify the dominant antibacterial agents within the cascade, radical scavenging experiments were subsequently conducted by selectively quenching specific ROS. CeO_2_/g‐C_3_N_4_ in a buffer solution containing Br^−^ was used as the model system, with isopropanol (IPA) employed to •OH [46], L‐methionine (Met) to quench HOBr [47], and catalase to decompose H_2_O_2_. As shown in Figure S16, the results showed that scavenging H_2_O_2_ with catalase completely suppressed the antibacterial activity, indicating that the process critically depends on piezoelectrically generated H_2_O_2_. When •OH was quenched by IPA, the antibacterial efficiency was retained at approximately 55%, whereas scavenging HOBr with Met led to a similar reduction in activity to around 38%. Notably, simultaneous quenching of both •OH and HOBr reduced the antibacterial efficiency to the background level, comparable to the H_2_O_2_‐depleted group. These findings demonstrate that both •OH and HOBr are key bactericidal ROS in the piezocatalytic system, with HOBr playing a particularly significant role in mediating the overall antibacterial effect. As illustrated in Figure 5f, CeO_2_/g‐C_3_N_4_ nanozymes can autonomously generate H_2_O_2_ in situ under hydrodynamic forces, sustaining their catalytic activity and enhancing ROS output. This self‐supplied substrate, combined with dual enzymatic mimicry, establishes a robust mechanistic basis for effective antibacterial action in seawater.

### 2.5 Antifouling Performance

**FIGURE 6:**
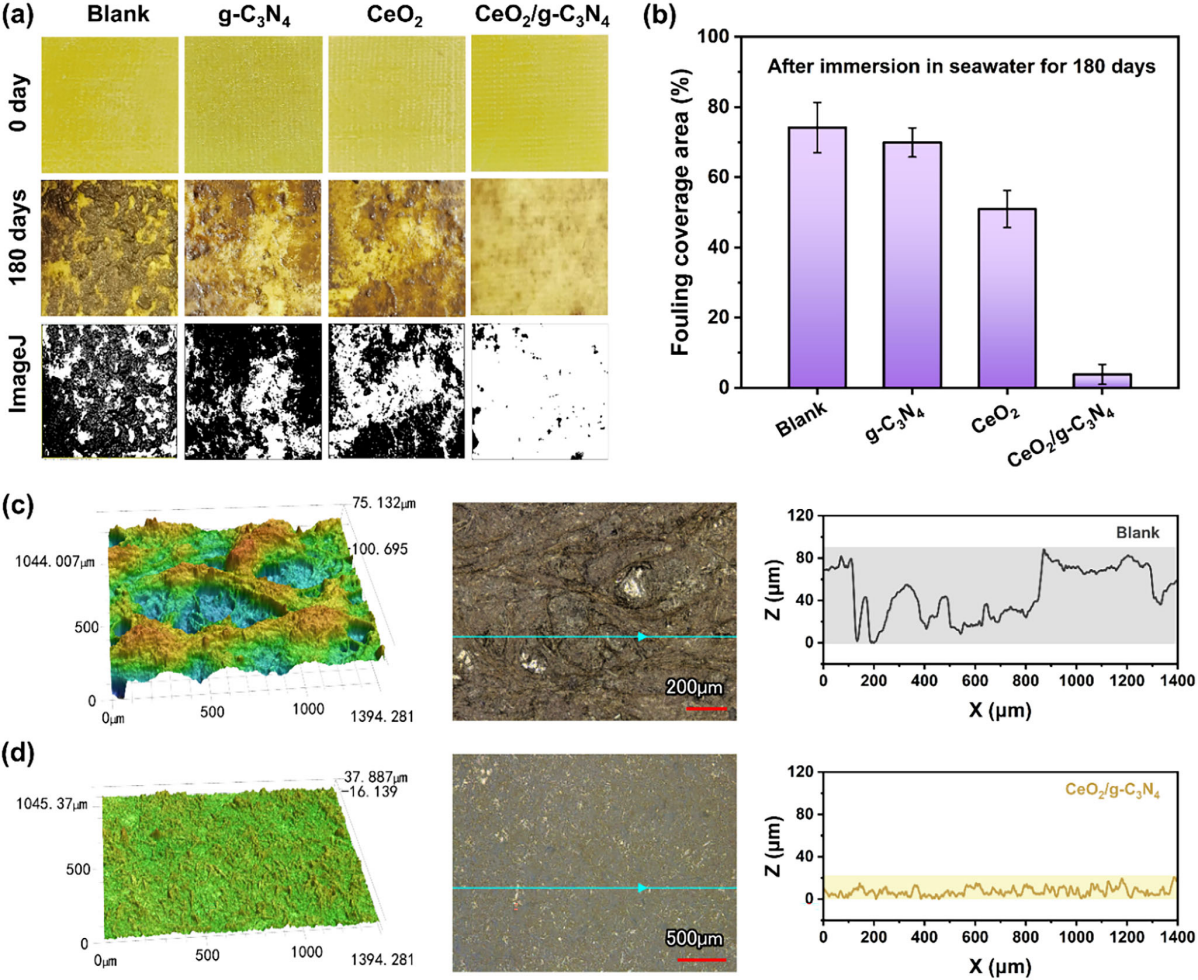
Marine antifouling performance. a) The surface of different samples after immersion in seawater for 6 months. b) Statistics of fouling coverage area of different surfaces after immersion in the Bohai Sea for 180 days. Error bars indicate standard deviations of three independent measurements. 3D surface topography, optical image, and surface profile of the c) blank epoxy panel and d) CeO_2_/g‐C_3_N_4_ nanozyme‐modified epoxy panel after 180 Days of Seawater Immersion.

Motivated by its outstanding indoor antibacterial performance, the long‐term marine antifouling efficacy of CeO_2_/g‐C_3_N_4_ nanozyme was rigorously evaluated through real‐sea trials conducted in the heavily polluted Bohai Sea (Figure S17). The fabrication procedure and basic characterization of the nanozyme‐incorporated coatings were summarized in Figure S18. Separately, the pre‐screening of CeO_2_/g‐C_3_N_4_ loading was presented in Figure S19, where the optimal content of 2.0 wt.% was selected based on antibacterial activity, adhesion, and surface wettability. To evaluate the long‐term antifouling performance of the coatings, a 180‐day field immersion test was conducted in the Bohai Sea. Epoxy resin panels were used as substrates, on which three types of nanozyme‑based coatings with the same loading of 2.0 wt.% were prepared: a CeO_2_/g‐C_3_N_4_ composite coating, a CeO_2_‑only coating, and a g‐C_3_N_4_‑only coating. Bare epoxy resin panels were employed as controls, and all panels were immersed in seawater simultaneously for testing. Biofouling progression was monitored via periodic photographic documentation, with all samples subjected to tidal forces, wave impacts, and other natural stressors during immersion (Figure S20). Figure 6a visually illustrates the fouling situation on different surfaces, and the exposed epoxy resin board shows severe biofouling, which verifies the high corrosiveness and severity of biofouling in the Bohai Sea area. In contrast, the CeO_2_/g‐C_3_N_4_‐coated panels exhibited remarkable antifouling performance, maintaining a fouling coverage of only 3.82% after 180 days, significantly lower than other controls (Figure 6b). The CeO_2_/g‐C_3_N_4_ nanozyme sustains ultralow biofouling coverage (<5%) throughout prolonged immersion, demonstrating exceptional stability and durability in marine environments. To further evaluate the antifouling performance of the coatings after long‐term marine exposure, laser microscopy was employed to characterize the surface morphology of epoxy panels immersed in natural seawater for 180 days. As shown in Figure 6c, the blank epoxy panel exhibited a rough and heterogeneous surface, with a fouling height of 82 µm, indicating severe biofouling and the accumulation of adherent residues. In contrast, the CeO_2_/g‐C_3_N_4_ nanozyme‐coated surface showed minimal biofouling, with a significantly reduced fouling height of only 21 µm (Figure 6d). These results demonstrate that the nanozyme coating effectively inhibits the attachment and growth of fouling organisms, preserving surface smoothness and integrity.

The sustained antifouling performance of the CeO_2_/g‐C_3_N_4_ nanozyme in marine environments stems from its integrated piezocatalytic and enzyme‐mimetic functionalities. Specifically, the piezoelectric response of the composite is activated by continuous wave and tidal forces, generating localized electric fields that induce the continuous in situ generation of H_2_O_2_, thereby overcoming the intrinsic scarcity of this substrate in natural seawater. The generated H_2_O_2_ is subsequently converted into ROS through the nanozyme's intrinsic HPO‐ and POD‐like catalytic activities, leading to effective suppression of biofouling. This self‐sustained catalytic process not only delivers long‐term antifouling efficacy but also avoids the use of toxic biocides, highlighting CeO_2_/g‐C_3_N_4_ as a promising environmentally friendly material for marine protection applications.

## 3 Conclusion

In summary, we have developed a bioinspired, self‐powered piezozyme by integrating piezoelectric g‐C_3_N_4_ with redox‐active CeO_2_, enabling an in situ H_2_O_2_‐supplying cascade for marine antifouling. The g‐C_3_N_4_ component efficiently converts ubiquitous low‐frequency mechanical stimuli, such as ocean waves, into H_2_O_2_ through piezocatalytic oxygen reduction, while CeO_2_ with dual HPO‐ and POD‐like activities subsequently transforms H_2_O_2_ into highly reactive species (•OH and HOBr) for effective bacterial suppression. This self‐powered antimicrobial pathway effectively overcomes the scarcity of H_2_O_2_ in natural seawater without any external chemical input. Laboratory antibacterial tests revealed >99.99% inactivation of both Gram‐negative and Gram‐positive bacteria, while a 180‐day marine field trial confirmed biofouling coverage below 5%, highlighting the coating's excellent durability and real‐world applicability. Beyond marine protection, the mechanically activated cascade nanozyme concept presented here opens new avenues for designing sustainable catalytic systems for energy‐limited or light‐deficient environments.

## 4 Experimental Section

### 4.1 Materials

Cerium(III) nitrate hexahydrate (Ce(NO_3_)_3_·6H_2_O, 99.5%) was purchased from Shanghai Macklin Biochemical Co., Ltd. Urea (CO(NH_2_)_2_, 99.5%), melamine (C_3_H_6_N_6_, 99%), ammonium chloride (NH_4_Cl, 99%), methyl methacrylate (MMA, 99%), butyl acrylate (BA, 99%), and γ‐methacryloxypropyltrimethoxysilane (KH‐570, 98%) were obtained from Shanghai Meryer Technology Co., Ltd. Heptadecafluorodecyl methacrylate (HDFMA, 97%) and ethyl acetate (EtOAc, AR) were provided by Shanghai Titan Scientific Co., Ltd. Azobisisobutyronitrile (AIBN, 98%) was purchased from Shanghai Aladdin Biochemical Technology Co., Ltd. Phenol red (PR, 95%), celestine blue (CB, 99%), disodium hydrogen phosphate dodecahydrate (Na_2_HPO_4_·12H_2_O, 99%), and potassium hydrogen phthalate (C_8_H_5_KO_4_, 99%) were obtained from Tianjin Heowns Biochem Technology Co., Ltd. Ammonium bromide (NH_4_Br, 99%), potassium iodide (KI, ≥99%), hydrogen peroxide (H_2_O_2_, 30%), and 3,3′,5,5′‐tetramethylbenzidine (TMB, 99%) were also purchased from Shanghai Aladdin Biochemical Technology Co., Ltd. Potassium dihydrogen phosphate (KH_2_PO_4_, 99%) and sodium chloride (NaCl, AR) were obtained from Tianjin Kemiou Chemical Reagent Co., Ltd. Agar powder (99%), peptone (99%), and yeast extract (99%) were supplied by Beijing Solarbio Science & Technology Co., Ltd. *Escherichia coli* (*E. coli*) and *Staphylococcus aureus (S. aureus)* were purchased from the China General Microbiological Culture Collection Center. All chemicals were used as received without further purification. All solutions were prepared using deionized water with a resistivity of 18.2 MΩ, unless otherwise stated.

### 4.2 Preparation of g‐C_3_N_4_, CeO_2_ and CeO_2_/g‐C_3_N_4_ Nanozymes

The synthesis protocol for the CeO_2_/g‐C_3_N_4_ nanozyme was modified from previously reported methods [48]. A solution was prepared by dissolving 2.5 g of Ce(NO_3_)_3_·6H_2_O in 30 mL of deionized water, followed by the sequential addition of 7.5 g urea, 2.5 g melamine, and 10 g NH_4_Cl. The mixture was ultrasonicated for 30 min to ensure complete dissolution. Subsequently, the solution was thoroughly impregnated and stirred in a water bath at 90°C to evaporate residual moisture. The resulting white solid was ground into a fine powder, transferred to a crucible, and calcined in a muffle furnace at 535°C for 3 h under ambient air with a heating rate of 5°C/min. The final product, obtained as a light‐yellow solid, was further pulverized into a homogeneous powder to yield the CeO_2_/g‐C_3_N_4_ nanozyme. In contrast, the pure g‐C_3_N_4_ and the pure CeO_2_ were obtained by the calcination method without adding Ce(NO_3_)_3_·6H_2_O or urea, melamine, and NH_4_Cl to the precursor mixture, respectively.

### 4.3 Piezocatalytic H_2_O_2_ Production Activity

The piezocatalytic H_2_O_2_ production was evaluated as follows. First, 80 mL of a 1 mg/mL catalyst material solution was prepared and stirred at 300 rpm in the dark for 30 min to achieve adsorption‐desorption equilibrium. The suspension was then subjected to ultrasonic vibration using an ultrasonic cleaner (40 kHz, 240 W, Beauty, Kunshan, China), simulating the mechanical energy from ocean waves or tides to activate the piezoelectric effect. During this process, samples were collected and filtered through a 0.22 µm membrane. A water circulation system maintained the entire reaction at room temperature, with all operations kept in complete darkness. For H_2_O_2_ quantification, the iodometric method was employed. Specifically, 1 mL of filtered sample was mixed with 1 mL of 0.4 M KI and 1 mL of 0.1 M C_8_H_5_KO_4_ solutions. The mixture was incubated for 30 min under ambient conditions, after which its absorbance at 350 nm was measured by UV‐vis spectroscopy. The H_2_O_2_ concentration was determined using the standard calibration curve.

### 4.4 Piezoelectric Enhanced HPO‐Like Activity Assays

The HPO‐like activity of g‐C_3_N_4_, CeO_2_, and CeO_2_/g‐C_3_N_4_ nanozymes was verified by determining the absorbance changes of PR at 434 nm and its brominated product at 590 nm using a UV‐visible spectrophotometer. In a typical test, the CeO_2_/g‐C_3_N_4_ nanozyme (100 µg mL^−1^) was added to an aqueous solution containing PR (50 µm) and Br^−^ (25 mm) in 3 mL of NaAc buffer (pH 4.5). The solution was treated with ultrasonic irradiation (40kHz, 240 W) for 120 min to simulate the mechanical energy from ocean waves or tides, and the absorbance was recorded as a function of reaction time. The same reactants under non‐ultrasound conditions were used as the control groups.

Validation of HOBr intermediate formation was performed by monitoring absorbance variations of CB at 640 nm using UV–vis spectrophotometry. In a typical assay, CeO_2_/g‐C_3_N_4_ nanozyme (100 µg mL^−1^) was introduced to an aqueous solution containing CB (50 µm) and Br^−^ (25 mm). The reaction system was subjected to ultrasonic irradiation (40kHz, 240 W) for 120 min, simulating mechanical energy, with absorbance recorded as a function of reaction time.

### 4.5 Piezoelectric Enhanced POD‐Like Activity Assay

The POD‐like activity of the sample was detected using TMB as the chromogenic substrate and its dependence on ultrasound was evaluated. In a typical experiment, a dispersion of CeO_2_/g‐C_3_N_4_ (100 µg/mL) was added to 3 mL of NaAc buffer (pH 4.0) containing TMB (0.5 mm). The reaction was conducted for 30 min under ultrasonic irradiation (40 kHz, 240 W) or under static conditions without ultrasound. The absorbance of the solution before and after the reaction was measured using a UV–vis spectrophotometer, and the difference in absorbance with and without ultrasound was recorded. The control groups were TMB alone, H_2_O_2_ + TMB, and CeO_2_/g‐C_3_N_4_ + H_2_O_2_ + TMB.

### 4.6 Antibacterial Performance of Nanozymes

The antibacterial activities of CeO_2_/g‐C_3_N_4_ nanozyme against *E. coli* and *S. aureus* were determined by standard plate counting method. CeO_2_/g‐C_3_N_4_ (200 µg mL^−1^), Br^−^ (5 mm), and H_2_O_2_ (250 nm) were combined with a fresh bacterial suspension (1 × 10^6^ CFU mL^−1^) in buffer solution within a sterile culture tube. The mixture was subjected to ultrasonic irradiation (40 kHz, 240 W) at 37°C for 120 min in the sonication group, while the non‐sonication group was maintained under identical conditions without ultrasonic treatment. Following appropriate serial dilution in sterile buffer solution, 100 µL aliquot was spread on LB agar plates. The bacterial colonies formed after 16 h static incubation at 37°C were counted. The bacteria treated with (1) Control (only bacterial), (2) g‐C_3_N_4_ + Br^−^, (3) CeO_2_ + Br^−^, (4) CeO_2_/g‐C_3_N_4_, and (5) CeO_2_/g‐C_3_N_4_ + Br^−^ were used as control groups. A comparison of antibacterial activity was made between the sonicated and non‐sonicated conditions to assess the effect of ultrasonic treatment on the antibacterial efficacy of the CeO_2_/g‐C_3_N_4_ nanozyme.

The antibacterial rate (*A*) was calculated using the following formula:

(1) $$A=\left(1-\frac{U_{1}}{U_{2}}\right)\;\times 100\%$$

where *U*_1_ and *U*_2_ represent the number of bacterial colonies in the blank group and the experimental group, respectively.

To elucidate the cascade reaction process of the CeO_2_/g‐C_3_N_4_ nanozyme under ultrasonic stimulation, the reaction system was synchronously sampled at different time points (5, 15, 30, 60, 90, and 120 min), and key intermediate species as well as antibacterial performance were monitored in parallel. The generation of H_2_O_2_ was quantitatively analyzed using the Titanium sulfate spectrophotometric assay, while •OH production was detected using a terephthalic acid (TPA) fluorescence probe. The formation of HOBr was monitored by a CB colorimetric assay, and bacterial viability at the corresponding time points was evaluated by the plate counting method.

### 4.7 Free Radical Inhibition Experiment

*E. coli* and *S. aureus* suspensions (≈ 1 × 10^6^ CFU mL^−1^) were prepared in buffer solution containing 5 mm NaBr. The CeO_2_/g‐C_3_N_4_ piezozyme was added at a concentration of 200 µg mL^−1^. Isopropanol (IPA, 50 mm), L‐methionine (5 mm), and catalase (20 U mL^−1^) were employed as scavengers for •OH, HOBr, and H_2_O_2_, respectively. All samples were subjected to ultrasonic stimulation (40 kHz, 240 W) at 25°C for 120 min. After treatment, 100 µL of the appropriately diluted suspension was spread onto LB agar plates and incubated at 37°C for 16 h. The surviving bacterial colonies were counted by the plate‐counting method, and the antibacterial efficiency was calculated based on the relative reduction in colony numbers.

### 4.8 Coatings Preparation

The coating was prepared via free‐radical copolymerization with slight modifications to reported methods [49]. Briefly, 15 mL of ethyl acetate was added to a 100 mL round‐bottom flask and purged with nitrogen at 70°C for 10 min. A monomer mixture containing 0.02 mol HDFMA, 0.02 mol MMA, 0.02 mol BA, 0.005 mol KH‐570, and 1 wt.% AIBN dissolved in 15 mL ethyl acetate was prepared and added dropwise over 1 h under nitrogen protection. The reaction was continued for an additional 2 h at 70°C to yield a transparent random copolymer solution.

### 4.9 Antifouling Performance of Nanozymes

During this 180‐day marine field test in the Bohai Sea, Tianjin, China, epoxy resin panels coated with various formulations were submerged 1.0 meter underwater. Each type of panel, with dimensions of 50 mm × 50 mm × 1 mm, had three replicates. The panels included: (1) blank epoxy resin panel (uncoated) and coatings containing (2) g‐C_3_N_4_, (3) CeO_2_, and (4) CeO_2_/g‐C_3_N_4_ nanozymes. Panels were periodically removed from the seawater at predetermined intervals, rinsed gently with seawater to remove loosely attached fouling organisms, and photographed to record fouling accumulation. The fouling area was then quantified using ImageJ software for accurate analysis.

### 4.10 Instrumentation

Transmission electron microscopy (TEM) images and energy‐dispersive X‐ray spectroscopy (EDS) were captured using a JEM‐F200 microscope (JEOL, Japan). Powder X‐ray diffraction (XRD) patterns were collected on a D8‐Focus diffractometer (Bruker AXS, Germany) equipped with Cu Kα radiation, scanning in the 2θ range of 5°–80° at a rate of 5°/min. Solid‐state ^13^C CP/MAS NMR spectra were recorded on a Bruker Avance Neo 400WB spectrometer (Germany) using a 4 mm MAS probe at room temperature. Fourier transform infrared (FT‐IR) spectroscopy was conducted using a Nicolet iS50 spectrometer (Thermo Scientific, USA) with an ATR accessory (resolution: 4 cm^−1^, 64 scans, range: 4000–400 cm^−1^). Raman spectra were recorded using a micro‐Raman spectrometer (inVia Reflex, Renishaw) with an excitation wavelength of 785 nm (diode laser) at a spectral resolution of 1 cm^−1^. X‐ray photoelectron spectroscopy (XPS) measurements were performed on a K‐Alpha+ spectrometer (Thermo Fisher Scientific, USA), and all spectra were calibrated to the C 1s peak at 284.8 eV. Thermogravimetric analysis (TGA) was performed on a HITACHI STA200 analyzer (Japan) under air atmosphere from 30°C to 800°C at a heating rate of 10°C/min. Electrochemical impedance spectroscopy (EIS) was conducted on a CHI 600E electrochemical workstation (CH Instruments, China) using a standard three‐electrode system. The piezo response force microscopy (PFM) was scanned on Bruker Dimension Icon to record the piezoelectric response. Kelvin probe force microscopy (KPFM) measurements were carried out on the same atomic force microscope (Bruker Dimension Icon) to characterize the surface potential distribution. Electron paramagnetic resonance (EPR) spectra were recorded on a Bruker EMXplus‐6/1 spectrometer (Bruker, Germany) to detect the characteristic signals of both reactive oxygen species (ROS) and oxygen vacancies. Ultraviolet‐visible (UV‐Vis) absorption spectra were recorded on a UV‐2700i spectrophotometer (SHIMADZU, Japan). 3D surface morphology of biofouling in real‐sea tests was observed using a laser microscope (KEYENCE VK‐X150). Contact angles were measured at room temperature using a goniometer via the sessile drop method (4 µL droplets), with average values derived from five distinct locations per sample. Coating adhesion strength was evaluated following ASTM D4541‐09 using a PosiTest AT‐A automatic adhesion tester (DeFelsko, USA): cylindrical aluminum studs (20 mm diameter) were affixed to coated epoxy panels and pulled at a constant rate of 0.2 MPa/s. Anti‐diatom adhesion was visualized using an optical microscope (Carl Zeiss, Germany).

## Conflicts of Interest

The authors declare no conflicts of interest.

## Supporting information




**Supporting File**: advs74423‐sup‐0001‐SuppMat.docx.

## Data Availability

The data that support the findings of this study are available from the corresponding author upon reasonable request.
