# Supplementary material for: Self‐Sustaining Piezozyme Platform for Antifouling via Mechanically Triggered Enzymatic Cascade
Source: Adv Sci (Weinh). 2026 Feb 20;13(23):e19278. doi: 10.1002/advs.202519278 (PMC13104074; doi:10.1002/advs.202519278)
Supplement: Supplementary file 1 — Supporting File: advs74423‐sup‐0001‐SuppMat.docx. [file ADVS-13-e19278-s001.docx]

Supporting Information

Self-Sustaining Piezozyme Platform for Antifouling via Mechanically Triggered Enzymatic Cascade

Jingyu Wang, Yanjun Yu, Rongxin Su* and Jiangjiexing Wu*

J. Wang, Prof. R. Su

State Key Laboratory of Chemical Engineering, Tianjin Key Laboratory of Membrane Science and Desalination Technology

School of Chemical Engineering and Technology

Tianjin University

Tianjin 300072 (China)

E-mail: [surx@tju.edu.cn](mailto:surx@tju.edu.cn)

Y. Yu

Technical Center for Safety of Industrial Products of Tianjin Customs

Tianjin 300457 (China)

Prof. R. Su, Prof. J. Wu

Key Laboratory of Ocean Observation Technology of Ministry of Natural Resources

School of Marine Science and Technology

Tianjin University

Tianjin 300072 (China)

E-mail: [wjjx1987@tju.edu.cn](mailto:wjjx1987@tju.edu.cn)

# Supplementary Figures and Tables


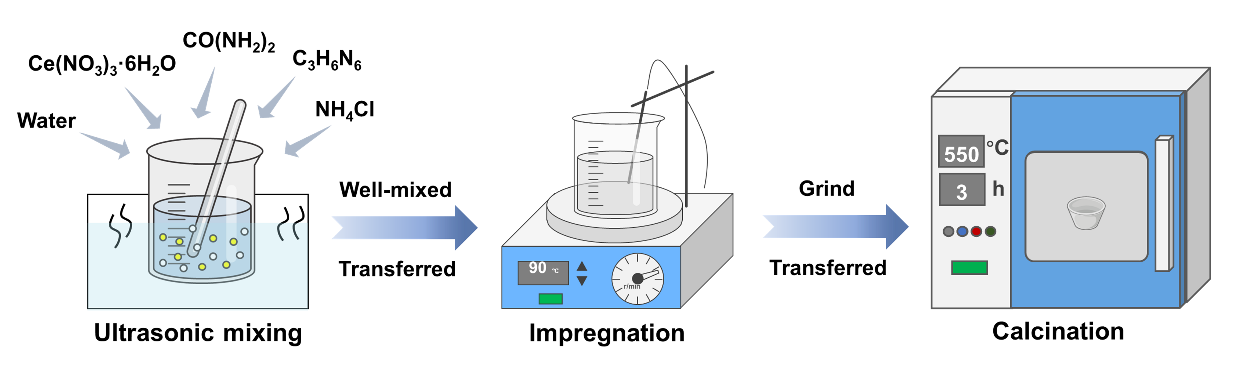


**Figure S1.** Schematic illustration of the preparation of the CeO_2_/g-C_3_N_4_.


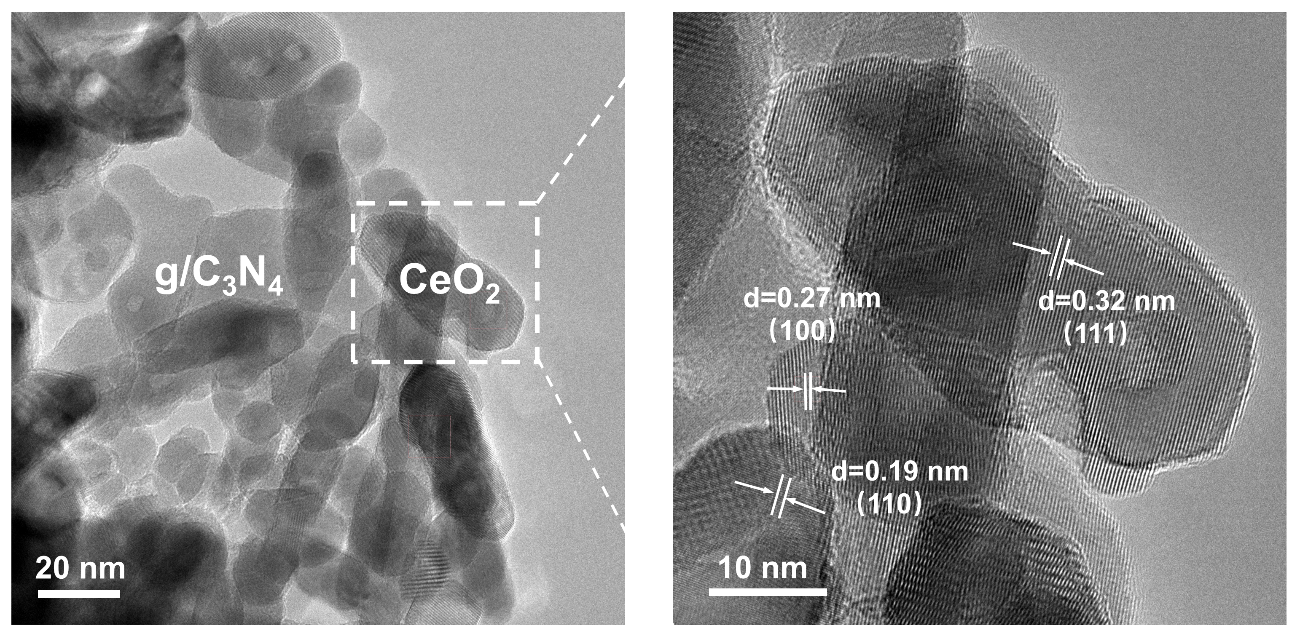


**Figure S2.** TEM and high-resolution TEM images of the CeO_2_/g-C_3_N_4_.


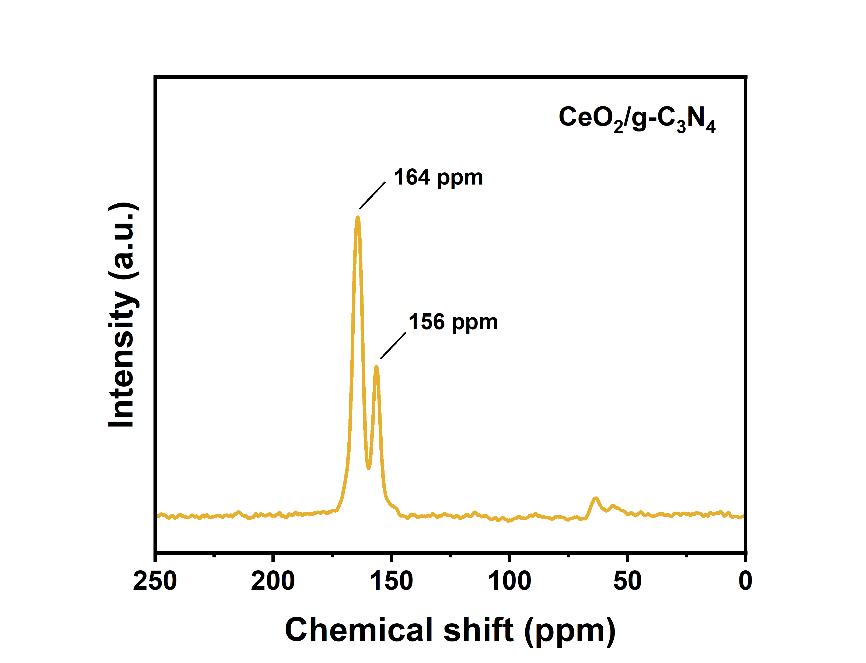


**Figure S3.** ^13^C NMR of CeO_2_/g-C_3_N_4_.


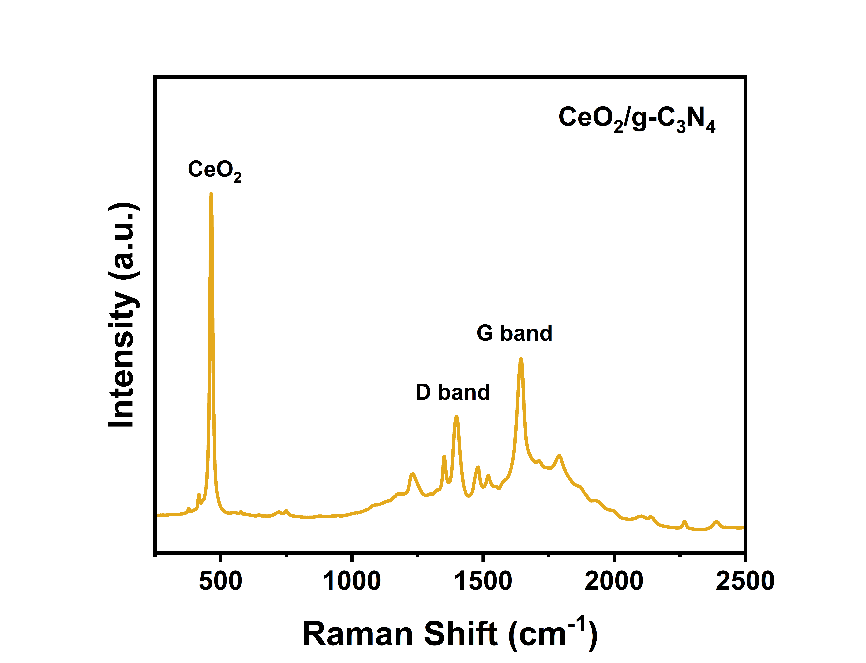


**Figure S4.** Raman spectrum of the CeO_2_/g-C_3_N_4_.


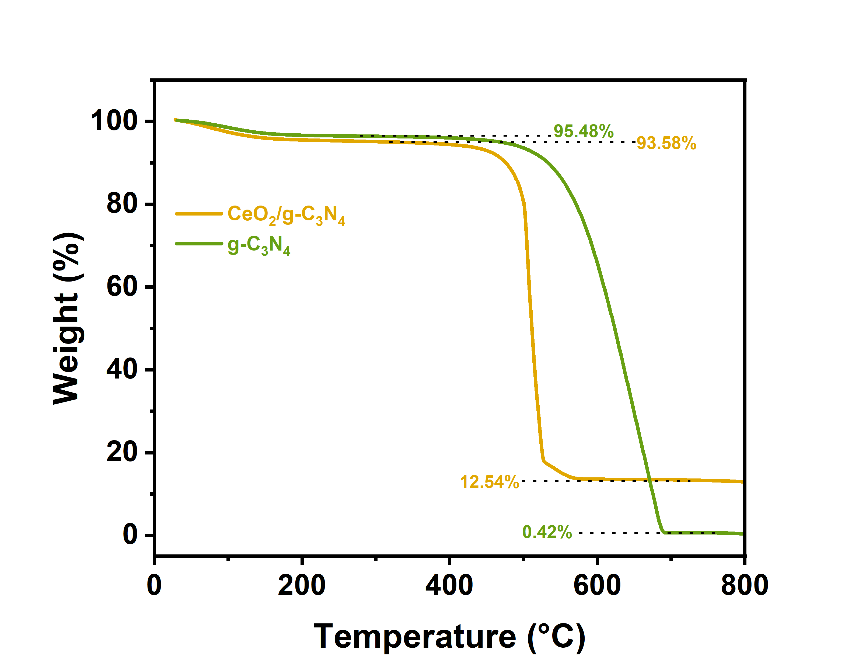


**Figure S5.** The TGA diagrams of the pure g-C_3_N_4_ and the CeO_2_/g-C_3_N_4_ nanozyme under air atmosphere.


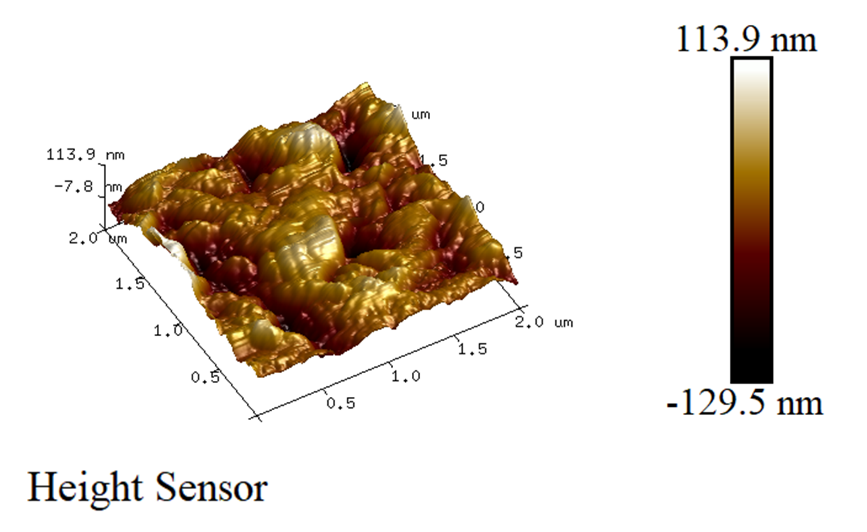


**Figure S6.** Surface topography and piezoresponse of CeO_2_/g-C_3_N_4_ observed via PFM.


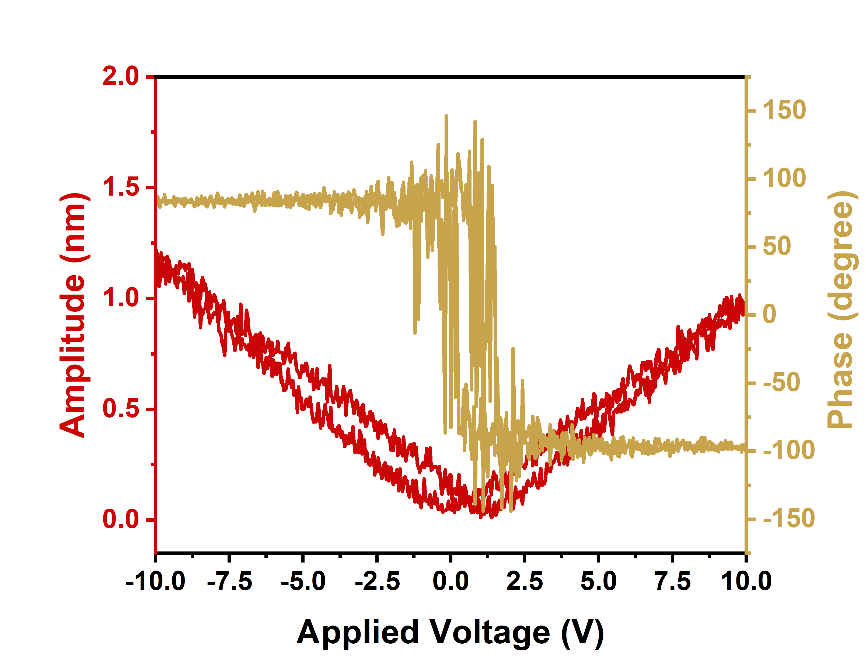


**Figure R7.** Piezoresponsive amplitude and phase curves of g-C_3_N_4_.


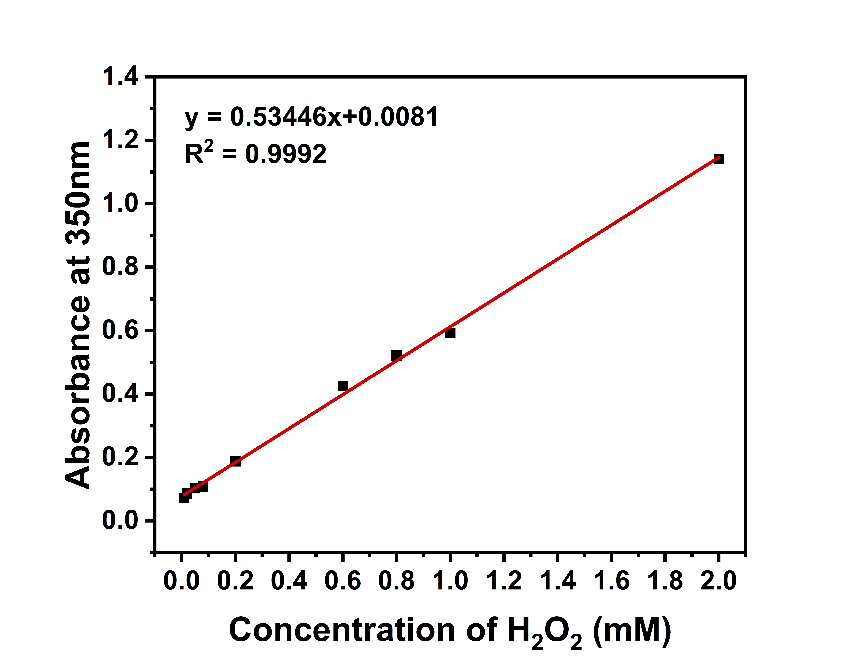


**Figure S8.** Standard calibration curve for H_2_O_2_ concentration in solution.


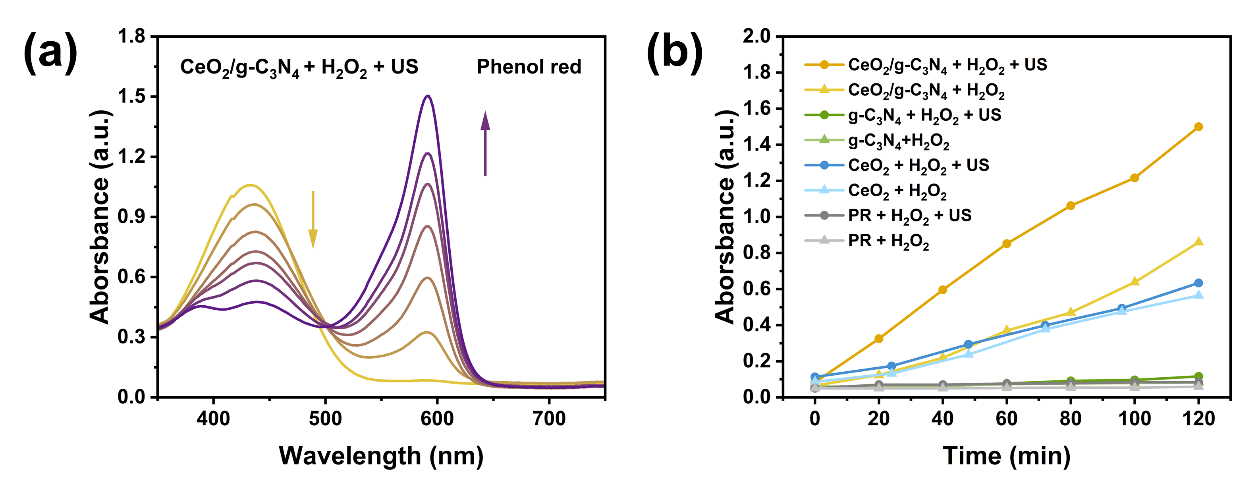


**Figure S9.** a) Time-dependent UV-Vis spectra illustrating the piezoelectric-enhanced HPO-like activities of CeO_2_/g-C_3_N_4_ under exogenous H_2_O_2_ conditions. b) Comparative analysis of HPO-like activities across experimental groups under ultrasonic irradiation and static conditions, with exogenous H_2_O_2_ supplementation.


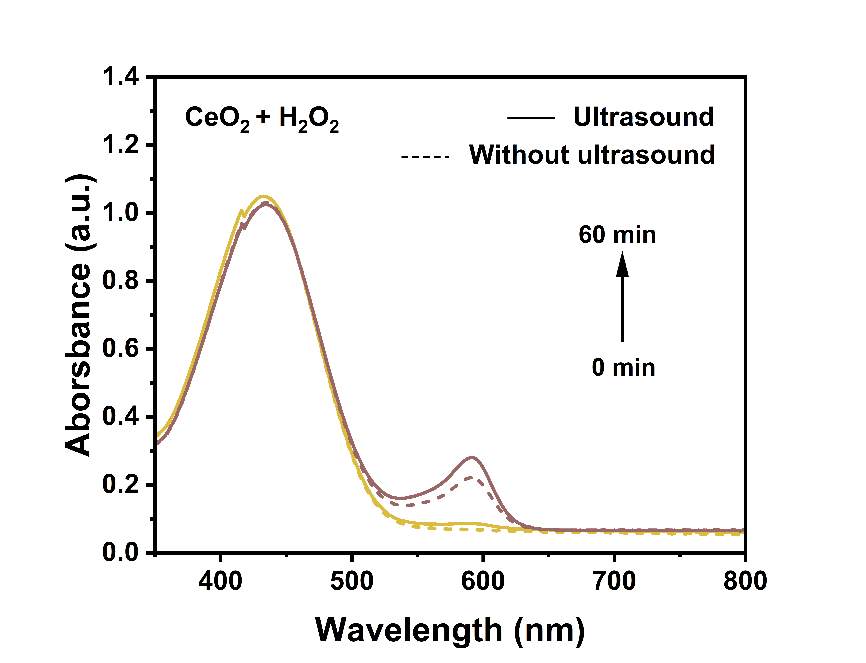


**Figure S10.** The HPO-like activities of CeO_2_ with exogenous H_2_O_2_ supplementation under ultrasonic irradiation and without ultrasonic irradiation.


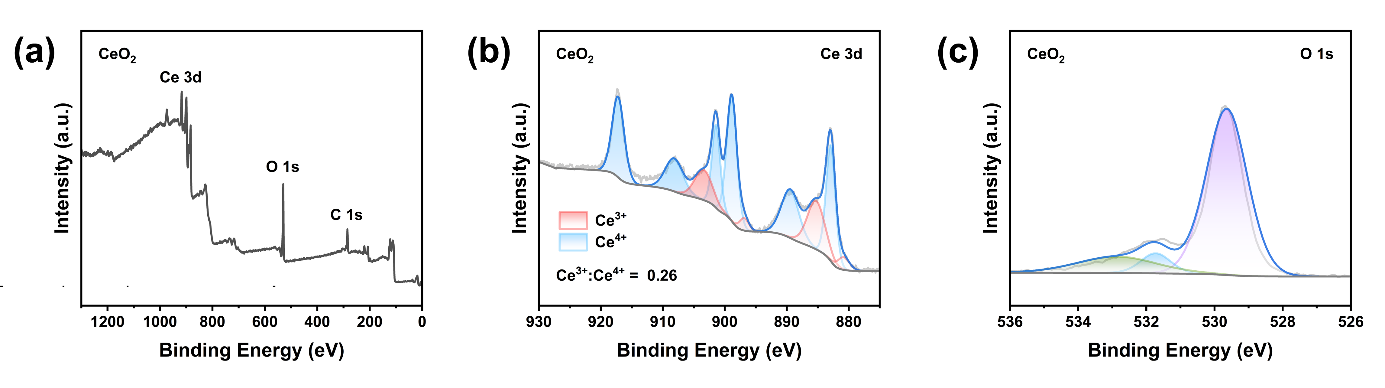


**Figure S11.** a) XPS survey spectrum of CeO_2_, high-resolution XPS spectra of Ce 3d b) and O 1s c) for CeO_2_.


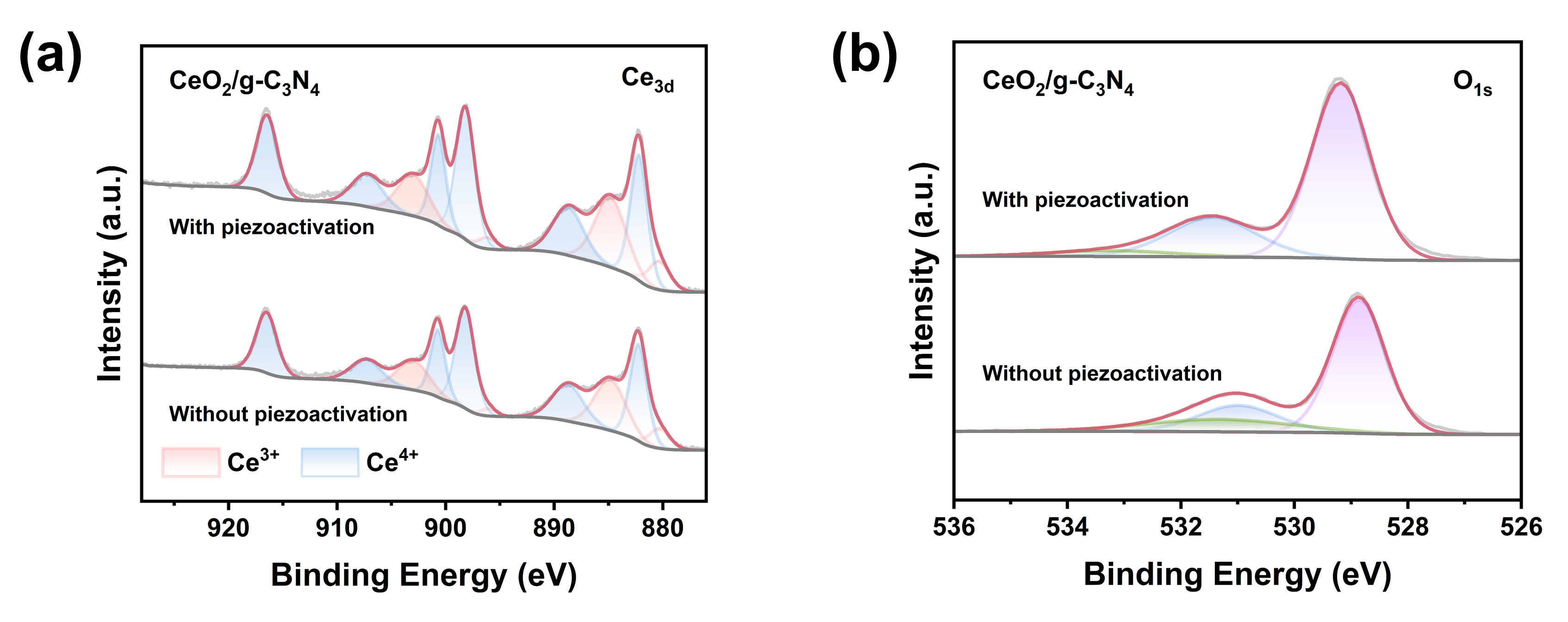


**Figure S12.** High-resolution a) Ce 3d and b) O 1s XPS spectra of the CeO_2_/g-C_3_N_4_ nanozyme before and after piezoelectric activation.


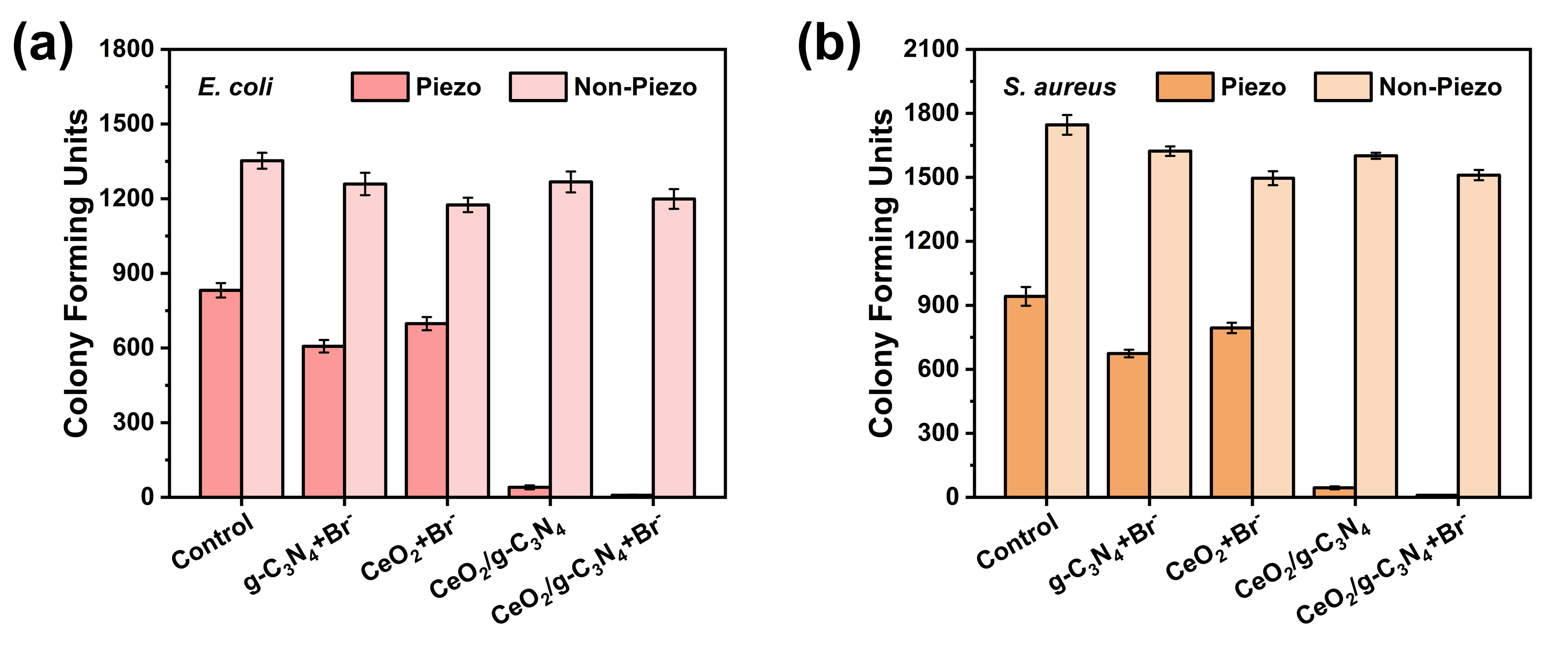


**Figure S13.** Counts of the number of colonies of a) *E. coli* and b) *S. aureus* with different treatments.


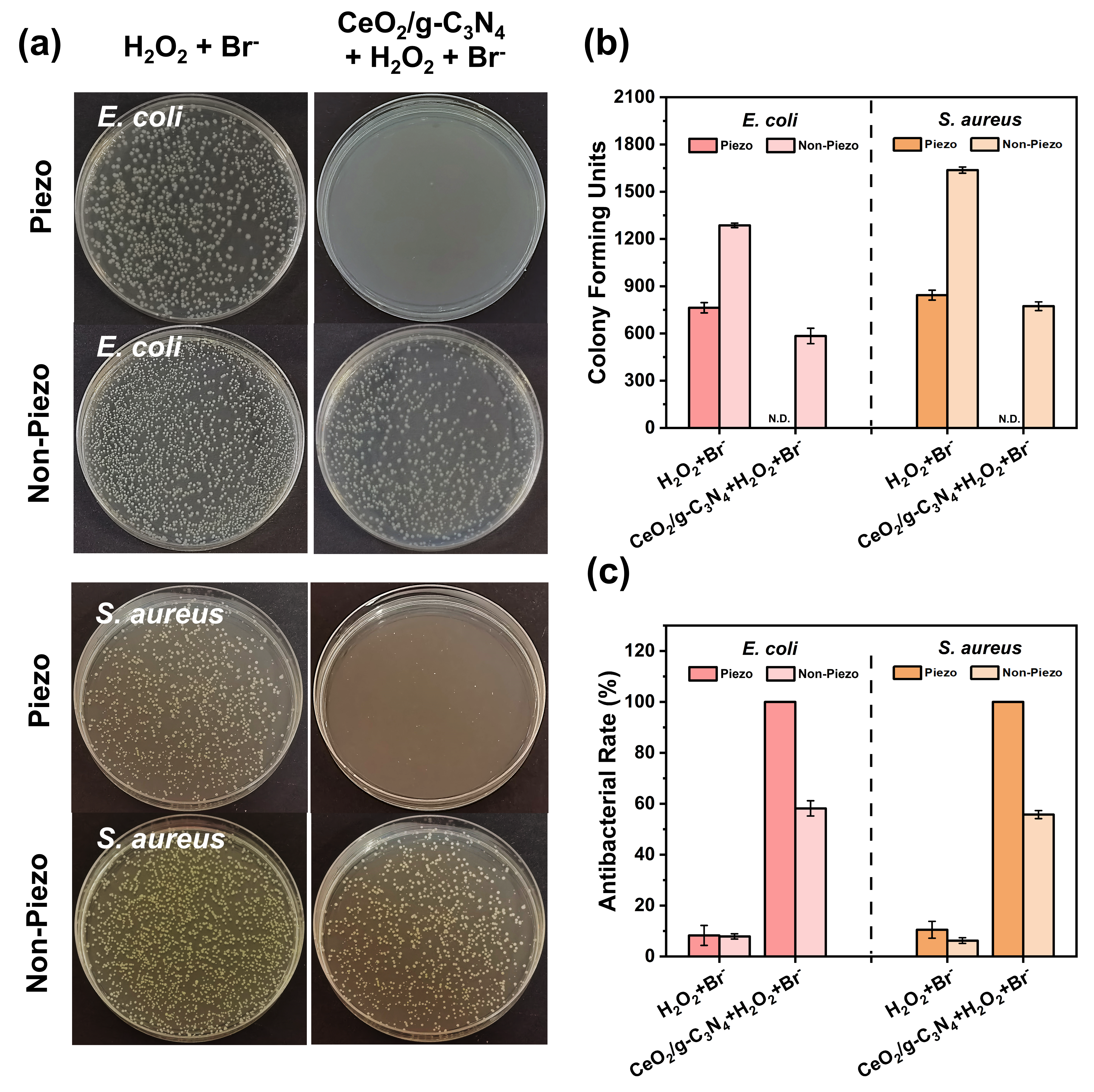


**Figure S14.** a) Digital photographs of colonies of *E. coli* and *S. aureus* treated with H_2_O_2_ + Br^−^ and CeO_2_/g-C_3_N_4_ + H_2_O_2_ + Br^−^. b) Counts of the number of colonies and c) Antibacterial rate of *E. coli* and *S. aureus* with different treatments. Error bars indicate standard deviations of three independent measurements.


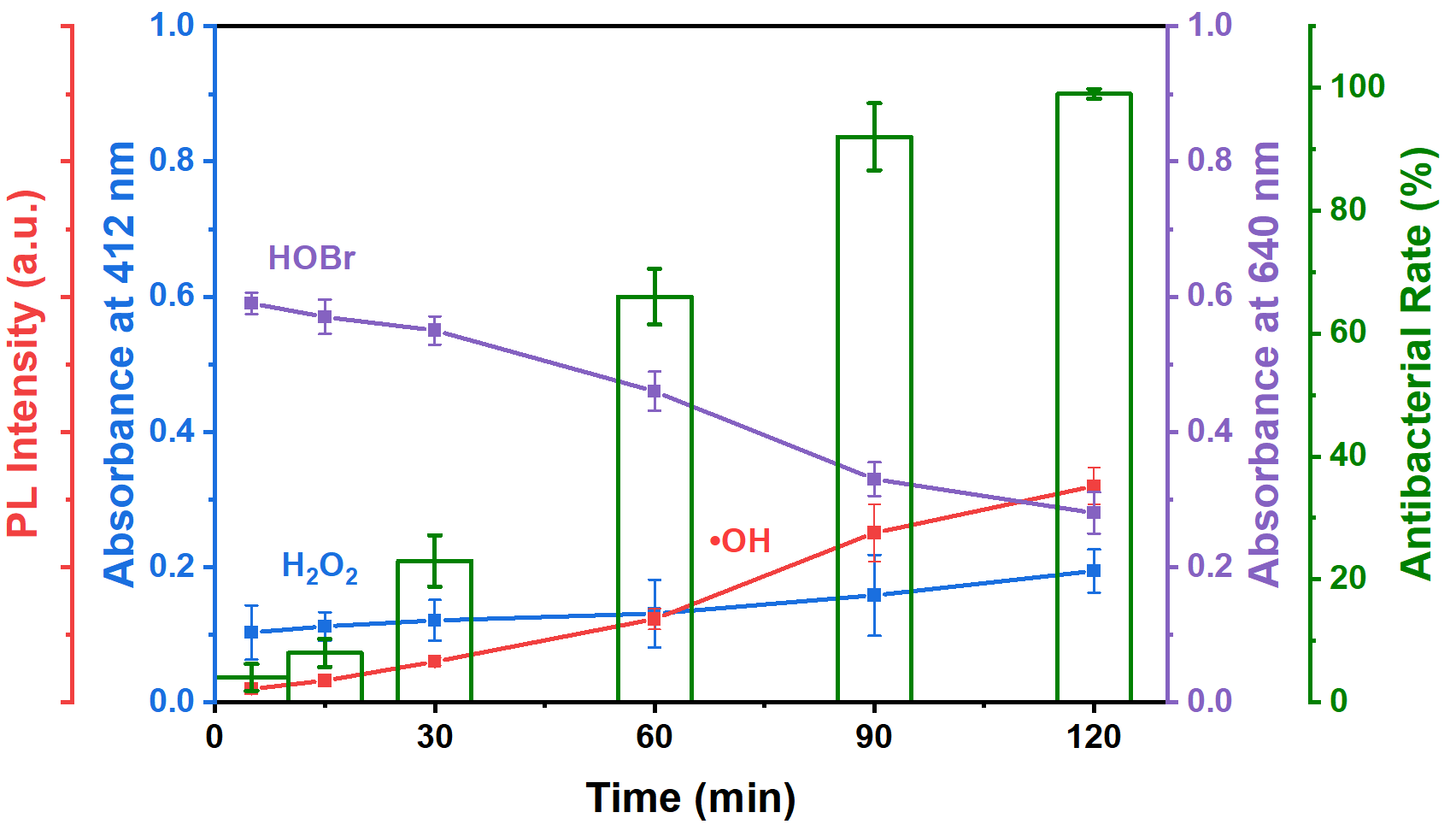


**Figure S15.** Time-dependent evolution of H_2_O_2_, •OH and HOBr concentrations, and antibacterial performance of CeO_2_/g-C_3_N_4_ nanozyme under piezoelectric excitation. (The concentrations were determined as follows: (i) H_2_O_2_ by titanium sulfate spectrophotometric assay (λ=412 nm), (ii) •OH by TPA fluorescence probe, (iii) HOBr by CB assay (monitoring absorbance decrease at ~640 nm). (iv) Antibacterial rate was assessed by the plate counting method.)


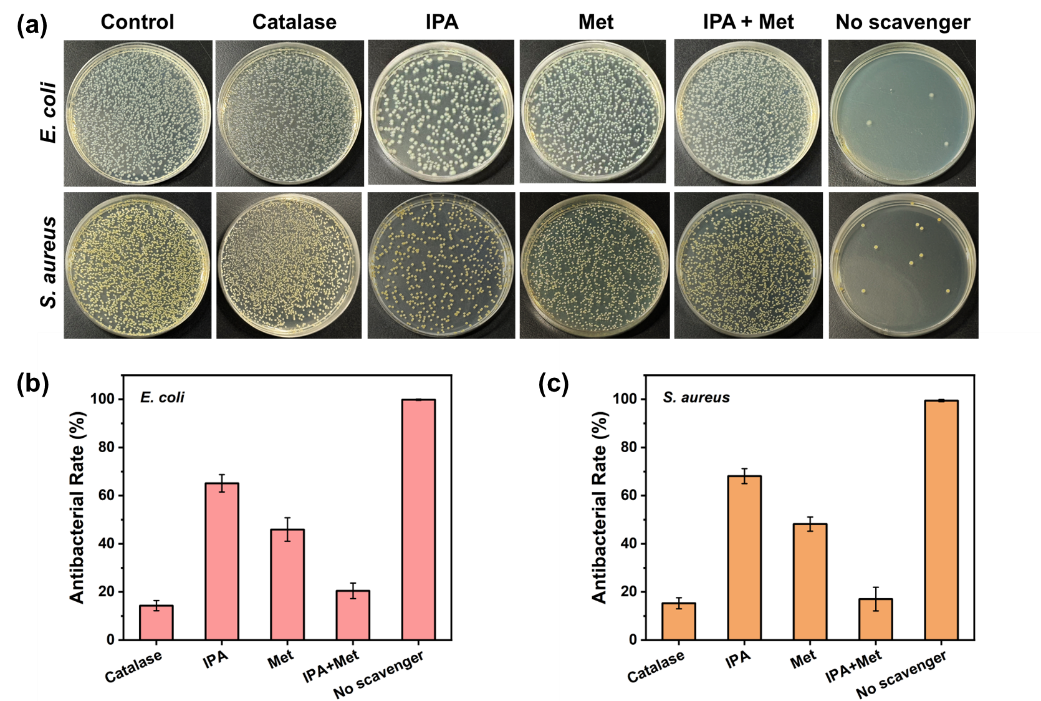


**Figure S16.** Free radical inhibition experiments for the CeO_2_/g-C_3_N_4_ piezozyme in the presence of different scavengers. a) Photographs of bacterial colonies, antibacterial rate of b) *E. coli* and c) *S. aureus*. Error bars indicate standard deviations of three independent measurements.


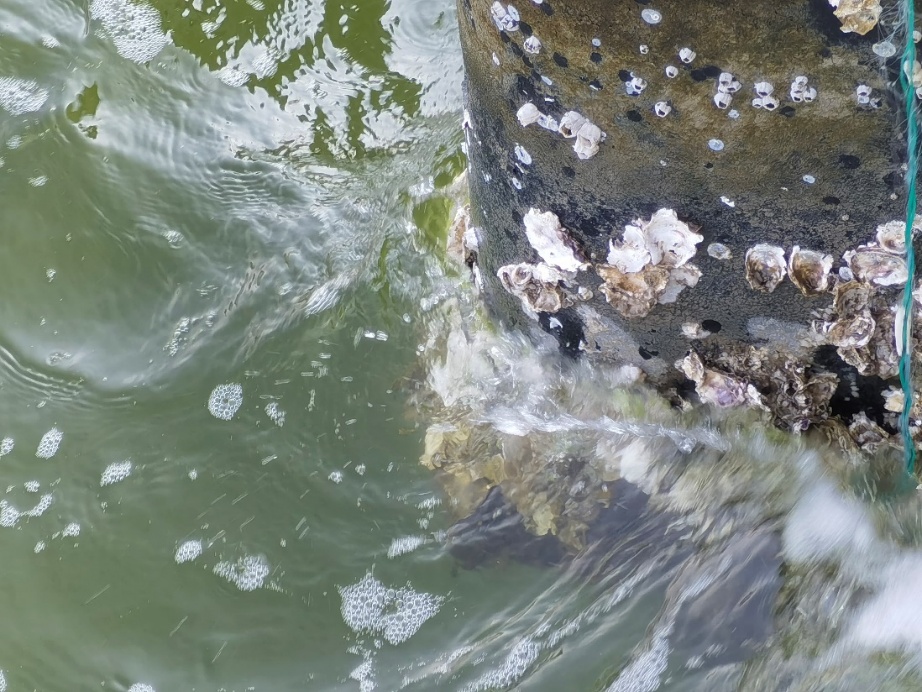


**Figure S17.** The growth of biological fouling in the field test environment.


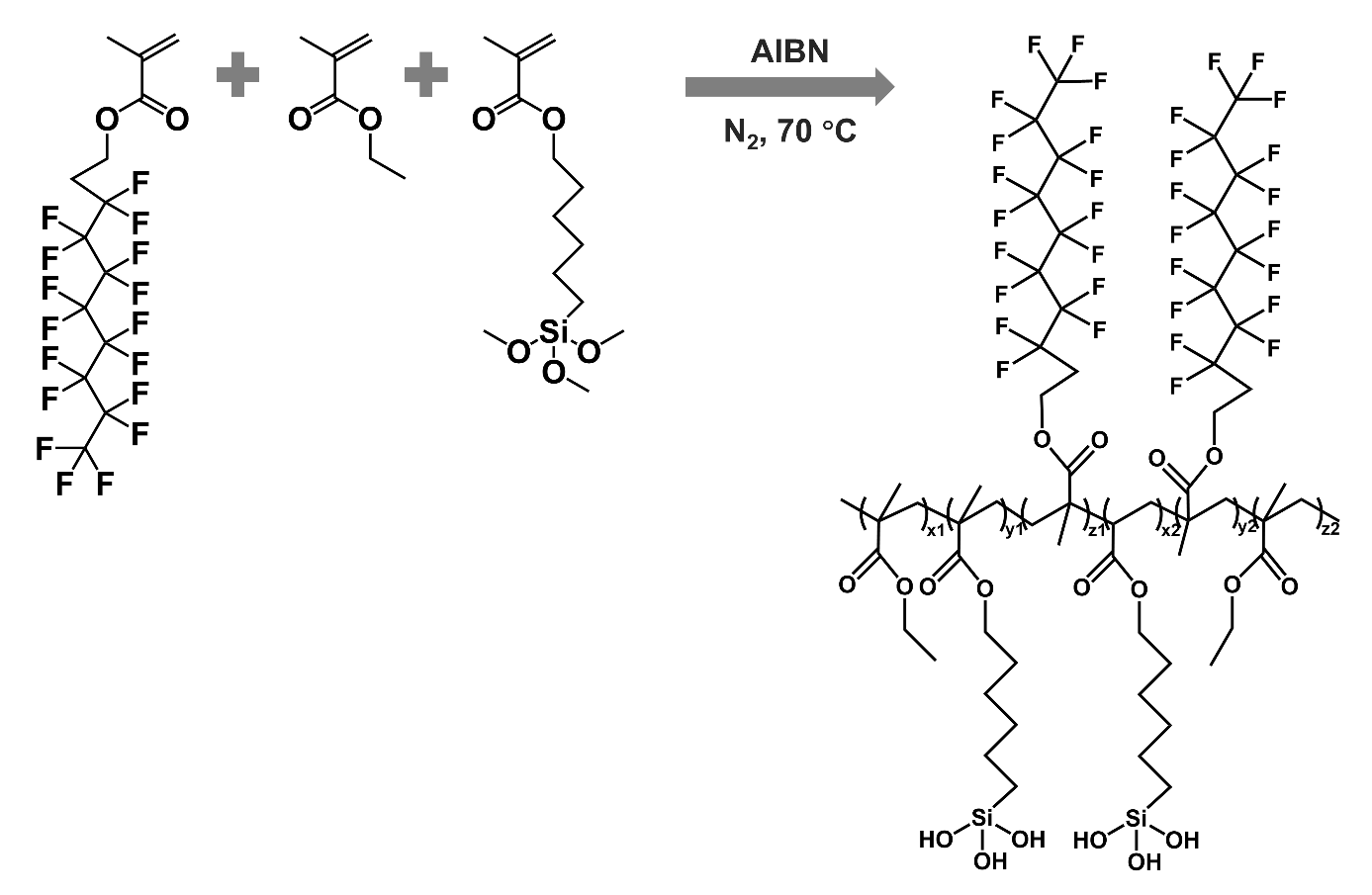


**Figure S18.** Schematic diagram of synthesis route of the coating polymer.


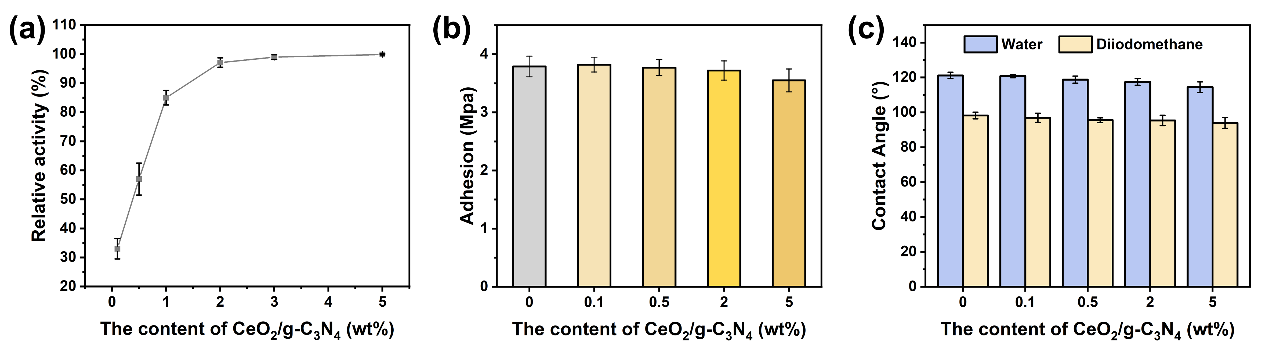


**Figure S19.** (a) Antibacterial performance, (b) adhesion, and (c) contact angles of coatings with varying CeO_2_/g-C_3_N_4_ loadings.


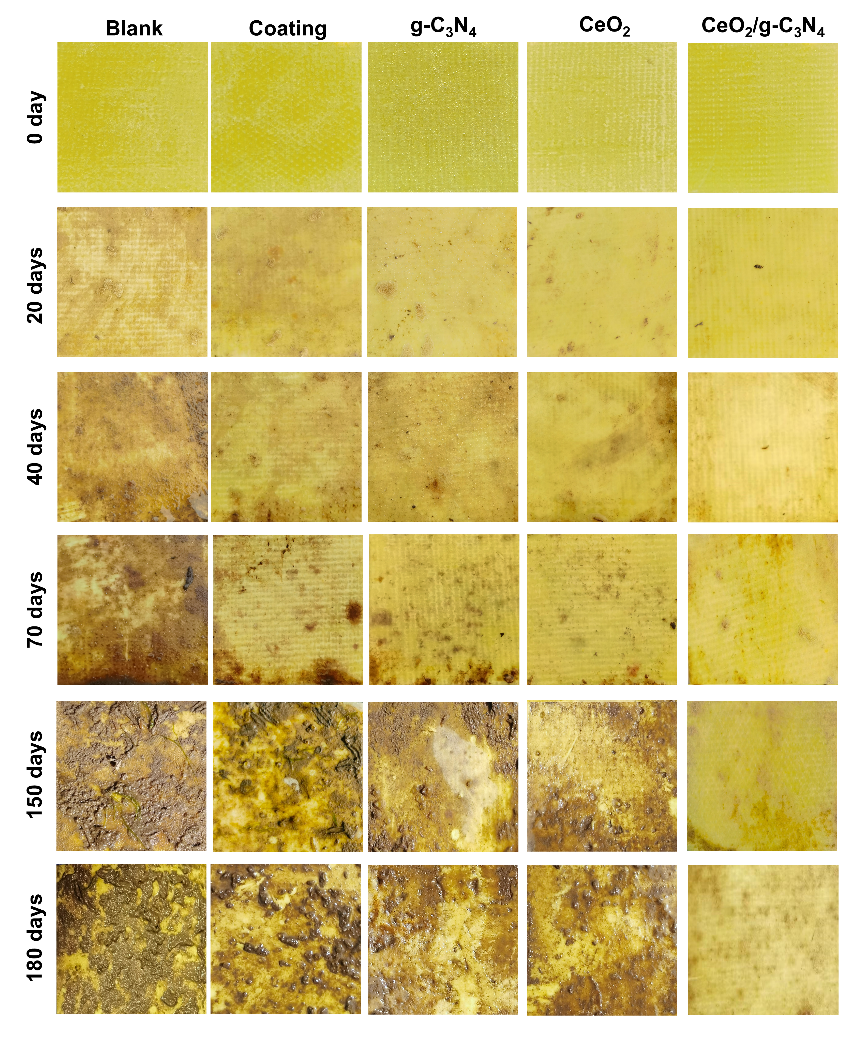


**Figure S20.** Surface appearance of different samples after 0, 20, 40, 70, 150 and 180 days of immersion in seawater.
